# Breast Cancer: Molecular Pathogenesis and Targeted Therapy

**DOI:** 10.1002/mco2.70404

**Published:** 2025-10-04

**Authors:** Md Abdus Samad, Iftikhar Ahmad, Mohammad Rashid Khan, Mohd Suhail, Torki A. Zughaibi, Fahad A. Al‐Abbasi, Khaled A. Alhosaini, Mohd Shahnawaz Khan, Ajoy Kumer, Shams Tabrez

**Affiliations:** ^1^ Department of Biochemistry Faculty of Science King Abdulaziz University Jeddah Saudi Arabia; ^2^ King Fahd Medical Research Center King Abdulaziz University Jeddah Saudi Arabia; ^3^ Department of Pharmacology and Toxicology College of Pharmacy King Saud University Riyadh Saudi Arabia; ^4^ Department of Medical Laboratory Sciences Faculty of Applied Medical Sciences King Abdulaziz University Jeddah Saudi Arabia; ^5^ Protein Research Chair Department of Biochemistry College of Sciences King Saud University Riyadh Saudi Arabia; ^6^ Department of Chemistry College of Arts and Sciences International University of Business Agriculture & Technology (IUBAT) Dhaka Bangladesh

**Keywords:** artificial intelligence, breast cancer subtypes, PI3K/Akt/mTOR inhibitors, PARP inhibitors, targeted therapy, tumor microenvironment

## Abstract

Breast cancer (BC) is the most prevalent cancer in women and remains the leading cause of cancer‐related mortality globally. Its development is influenced by multiple factors, including genetics, environmental, aging, and modulation of various signaling pathways. The heterogeneity of BC together with the emergence of treatment resistance and recurrence have prompted researchers to explore and develop new therapeutic approaches. Recently, oncology research has primarily focused on the development of targeted therapies against molecular abnormalities in BC. These therapies include monoclonal antibodies, tyrosine kinase inhibitors, antibody–drug conjugates, PI3K/Akt/mTOR pathway inhibitors, CDK 4/6 inhibitors, PARP inhibitors, antiangiogenic agents, and various other targeted drugs. Immunomodulatory strategies, including immune checkpoint inhibitors (anti‐PD‐1/PD‐L1), CTLA‐4 blockers, adoptive T‐cell therapy, and cancer vaccines, stimulate immune response against cancer cells. Epigenetic therapies like DNMT and HDAC inhibitors have also shown promise in BC treatment. This review highlights how innovative approaches like targeting intratumoral heterogeneity, liquid biopsy for resistance mutation detection, bypass mechanisms (*FGFR1* activation following CDK4/6 inhibition), artificial intelligence‐based drug discovery, patient‐derived organoids, and adaptive trial designs are shaping BC treatment. By combining molecular insights with precision therapeutics, these advancements offer significant potential to address resistance, improve efficacy, and enhance patient outcomes.

## Graphical abstract

Dysregulation signaling pathways in various breast cancer subtypes and their targeting using different inhibitors.

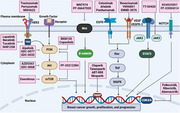

## 1 Introduction

Breast cancer (BC) is the most frequently diagnosed malignancy in women and has a high incidence rate. It is the second most common cancer globally and remains the most prevalent type affecting women [1, 2]. In 2024, approximately 310,720 new cases of invasive BC were diagnosed in women in the United States, with an estimated 42,250 related deaths and the incidence of the disease continues to increase annually [3]. In 2022, approximately 2.3 million new cases and 670,000 deaths were recorded worldwide [4, 5]. Projections suggest that by 2050, the number of new cases could increase to 3.2 million annually, with deaths reaching 1.1 million per year [5].

BC is a multifaceted and diverse disease influenced by various risk factors including age, genetic changes, smoking, alcohol consumption, diet, obesity, early onset of menstruation, and late menopause [6, 7]. Despite extensive research, spanning from preclinical to clinical studies, it continues to pose a significant challenge for healthcare professionals, as the precise mechanisms underlying its development remain unclear [8]. Researchers have thoroughly examined the molecular and biological characteristics of BC to better understand its development and the underlying mechanisms. This includes the investigation of specific gene mutations, oncogenes, tumor suppressor genes, and various downstream signaling pathways [9, 10]. Notably, pathways such as estrogen receptor (ER), human epidermal growth factor receptor 2 (HER2), PI3K/Akt/mTOR, Wnt/β‐catenin, Janus kinase 2 (JAK2)/signal transducer and activator of transcription 3 (STAT3), Notch, and Hedgehog (Hh) signaling have been analyzed to elucidate their roles in BC progression [11, 12]. These pathways contribute to tumor growth, regulate cell differentiation, suppress apoptosis, facilitate angiogenesis, drive metastasis, and contribute to drug resistance [13, 14].

Scientists have applied various techniques for the treatment of BC, such as local treatments, surgery, chemotherapy, endocrine therapy (ET), radiation therapy, and other strategies. However, these approaches have not fully resolved the issue due to factors, such as tumor heterogeneity, drug resistance, BC recurrence, and adverse side effects [15, 16]. Recently, scientists have focused on developing therapies that specifically target certain conditions to address treatment difficulties and improve patient outcomes. The idea of targeted therapy was first introduced in the 1890s by German scientist Paul Ehrlich, who proposed Magic Bullet Theory. He envisioned a treatment that would selectively target diseased cells while causing no harm to healthy cells, thereby eliminating toxicity [17]. Although this concept was initially applied to infectious diseases, it was not immediately utilized in cancer treatment because of the limited understanding of cancer etiology and biology at the time [18]. However, with advancements in research, this approach has since been extended to cancer therapies, including BC [19]. Target therapy improves the efficacy of drugs while reducing adverse reactions compared with conventional chemotherapy, as it selectively targets cancer cells without significantly harming healthy tissues [20]. It also helps to overcome drug resistance [21]. Furthermore, targeted therapies play a crucial role in metastatic BC (MBC) and personalized medicine, allowing tailored treatments to improve patient outcomes [22].

BC‐targeted therapies involve the use of specific drugs or substances designed to hinder disease progression by blocking the key processes necessary for cancer cell survival, growth, invasion, metastasis, migration, and angiogenesis [23, 24]. Various compounds, including HER2‐targeted monoclonal antibodies (mAbs), PI3K inhibitors, tyrosine kinase inhibitors (TKIs), Akt inhibitors, and mTOR inhibitors, selectively interfere with various signaling cascades that regulate cell growth, differentiation, proliferation, survival, and apoptosis [25, 26]. These agents, whether used individually or in combination, have significantly improved treatment outcomes in BC therapy [27]. In addition, cyclin‐dependent kinase 4/6 inhibitors (CDK 4/6), poly ADP‐ribose polymerase (PARP), vascular endothelial growth factor receptor (VEGFR), and epidermal growth factor receptor (EGFR) are well‐recognized therapeutic targets that have become central to the development of drugs for BC treatment [28, 29]. Patients undergoing targeted therapy often experience common side effects, such as nausea, vomiting, anemia, abnormal pain, fatigue, headaches, diarrhea, and skin rashes [30, 31]. Therefore, it is crucial to manage the toxicity associated with these therapies to ensure improved patient management and treatment outcomes.

In this review, we explore the molecular pathogenesis of BC and various targeted therapies used for its treatment. We discuss the effectiveness and safety of HER2‐targeted therapies, CDK4/6 inhibitors, PI3K/Akt/mTOR inhibitors, PARP inhibitors, and angiogenesis inhibitors, along with other therapeutic approaches in clinical practice. This review also discusses the molecular basis of drug resistance and therapeutic failure, particularly highlighting the difficulties associated with the triple‐negative BC (TNBC) and HER2^+^ subtypes. Finally, we compile recent clinical findings and propose future directions for designing therapies and planning personalized treatments. By employing this organized approach, we aim to improve clinicians’ comprehension and guide researchers in identifying new molecular targets and therapeutic approaches for various BC subtypes.

## 2 Overview of BC Pathogenesis

### 2.1 BC Initiation and Progression

**FIGURE 1:**
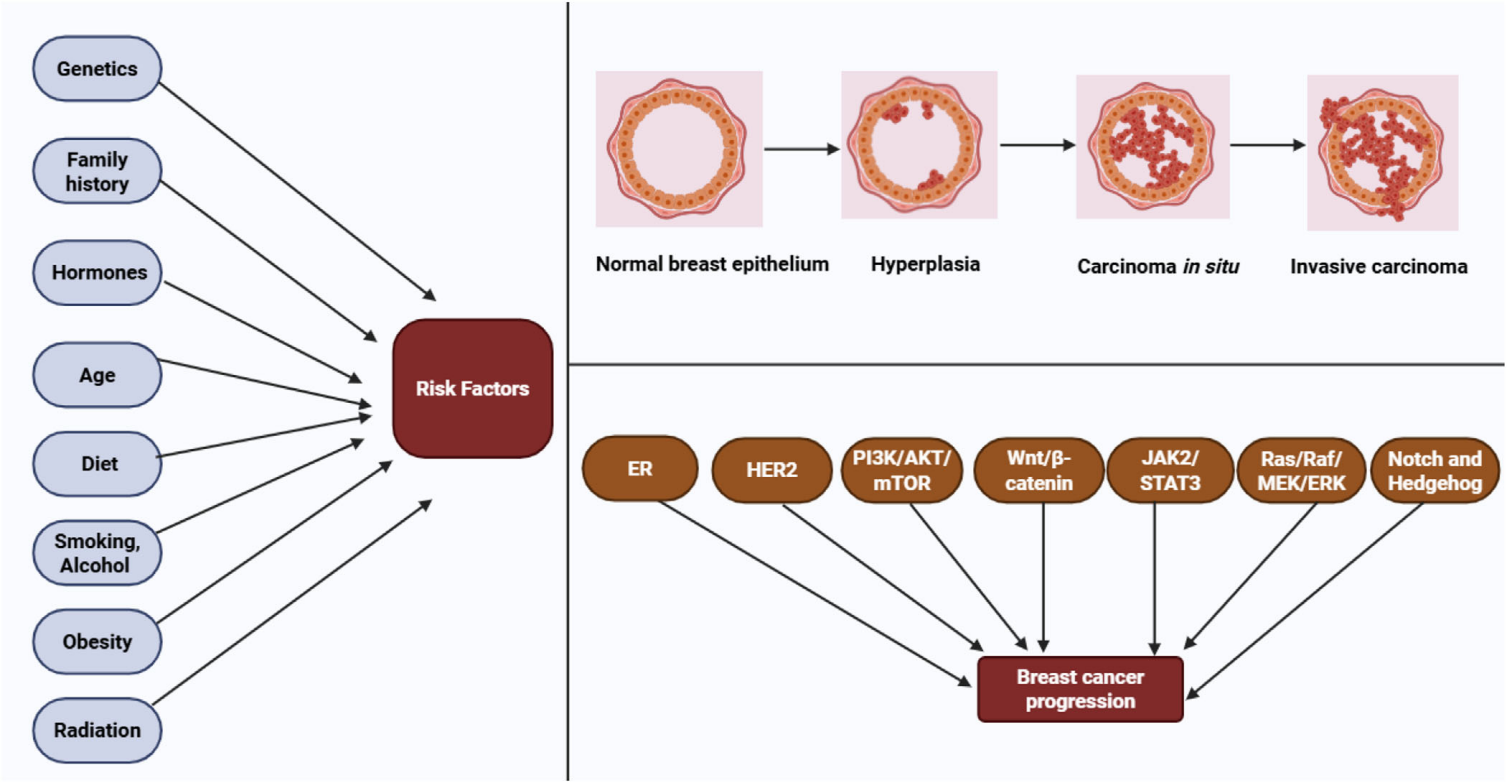
Overview of breast cancer initiation and progression. Breast cancer pathogenesis is influenced by various molecular and environmental risk factors. It begins through the mutation of oncogenes, tumor suppressor genes, and dysregulation of key cell signaling pathway, which lead to normal breast epithelium to hyperplasia. After hyperplasia, cells undergo to preinvasive stage (carcinoma in situ) and eventually to invasive carcinoma. [Created using BioRender.com.]

BC initiation and progression are complex, multistep processes that are influenced by various molecular and environmental factors [32]. These risk factors include genetics, family history, hormones, age, diet, smoking, alcohol consumption, obesity, radiation exposure, and others [33, 34]. It starts with abnormal cell growth in the breast tissue and can evolve through different stages, eventually leading to invasive disease and metastasis [35]. Typically, BC begins with mutations in the DNA of healthy breast cells. These mutations can include gain‐of‐function (GoF) mutations in oncogenes, such as *HER2* and *PI3KCA* (phosphatidylinositol‐4,5‐bisphosphate 3‐kinase catalytic subunit alpha), or loss‐of‐function mutations in tumor suppressor genes, such as *BRCA1* (BC type 1), *BRCA2*, *ERS1* (ER gene), *ATM* (ataxia‐telangiectasia mutated), *TP53* (tumor protein p53), *PALB2* (partner and localizer of *BRCA2*), *CHEK2* (checkpoint kinase 2), *PTEN* (phosphatase and tensin homolog), *STK11* (serine/threonine kinase 11), and *NF1* (neurofibromatosis type 1) [36, 37]. These genetic alterations can cause normal breast cells to become cancerous, triggering uncontrolled cell growth [38]. As the disease progresses, the affected cells may progress to hyperplasia, an increase in cell number, which can then lead to atypical hyperplasia, a condition linked to a greater risk of cancer progression [39]. During the early stages, the tumor is typically localized and confined to the ducts or lobules of the breast, forming conditions, such as ductal carcinoma in situ or lobular carcinoma in situ, both of which are considered pre‐invasive stages as the cancer has not spread beyond the breast [40]. Additionally, several molecular pathways, such as ER, HER2, PI3K/Akt/mTOR, Wnt/β‐catenin, JAK2/STAT3, Ras/Raf/MEK/ERK, Notch, and Hh, play significant roles in promoting BC progression by increasing cancer cell survival, proliferation, metastasis, angiogenesis, and resistance to therapy [41, 42]. Figure 1 illustrates the initiation and progression of BC influenced by different risk factors.

### 2.2 Molecular Typing of BC

BC pathogenesis is highly heterogeneous and involves various molecular subtypes that differ significantly in clinical behavior, therapeutic responsiveness, and prognosis [34, 43]. BC is typically divided into three primary categories based on hormone receptor (HR) status. The first type includes tumors that express either ER or progesterone receptor (PR). The second type involves the expression of HER2, with or without the expression of ER and PR. The third type is TNBC, which is characterized by the absence of ER, PR, and HER2 expression [44]. In addition, BC has been classified into intrinsic molecular subtypes through gene expression profiling. These subtypes include luminal A (ER^+^PR^+^, Ki‐67^low^, expression of luminal genes: *ESR1*, *FOXA1, GATA3*, and *XBP1*), luminal B (ER^+^, Ki‐67^high^ lower expression of luminal genes: *FOX1* and *PGR*), HER2‐enriched (HER2^+^, moderate expression of luminal genes), TNBC, or basal‐like (high expression of *EGFR* and low expression of the luminal A genes) [45, 46]. A summary of these subtypes with their distinct characteristics and molecular features is presented in Table 1.

**TABLE 1:** Breast cancer subtypes with their distinct characteristics and molecular features.

Features	Luminal A	Luminal B	HER2^+^	TNBC	References
Receptor status	ER^++^, PR^++^, HER2^−^	ER^+^, PR^+^, HER2^+/−^	ER^−^, PR^−^, HER2^+^	ER^−^, PR^−^, HER2^−^	[43]
Gene expression	High expression of luminal genes	Lower expression of luminal genes	Moderate expression of luminal genes	Low expression of luminal A genes, high expression of *EGFR*	[45]
Proliferation (Ki‐67)	Ki‐67^low^	Ki‐67^high^	Ki‐67^high^	Ki‐67^high^	[47]
Percentage of instances	60%	10%	20%	10%	[48]
Aggressiveness	Less aggressive	More aggressive	More aggressive	Highly aggressive	[49]
Prognosis	Favorable	Moderate	Moderate	Poor	[50]
Therapy	Hormone therapy	Hormone therapy	HER2^−^ targeted therapy	Chemotherapy	[51]

## 3 Molecular Pathogenesis: From Genomic Alterations to Hallmarks of Cancer

The molecular pathogenesis of BC is a highly intricate process driven by genetic mutations, hormonal dysregulation, and immune system aberration [52]. These factors collectively lead to the emergence of key cancer hallmarks such as sustained proliferative signaling, evasion of growth suppressors, resistance to cell death, replicative immortality, induction of angiogenesis, activation of invasion and metastasis, immune evasion, metabolic reprogramming, and therapeutic resistance [53]. Furthermore, the tumor microenvironment (TME) influences BC progression through dynamic interactions with tumor cells, immune cells, stromal cells, and various nonmalignant elements [54].

### 3.1 Genomic Landscape

The genomic landscape of BC is complex and highly heterogeneous, with variations in gene mutations, copy number alterations (CNAs), genomic alterations, and other genomic changes [55]. Studies have focused on identifying the most common mutated genes and examining how mutation types differ across various age and ethnic populations [56, 57].

The *HER2* gene, also known as *ErbB2* gene, encodes a receptor tyrosine kinase (TK) and is activated by extracellular growth factors, triggering intracellular signaling cascades involved in cell growth, survival, and embryonic development [58]. When *HER2* is mutated or overexpressed of it leads to abnormal signaling, disrupts cellular homeostasis, and contributes to oncogenesis [59]. *HER2* mutations are found in approximately 2–5% of patients and function as oncogenic drivers, leading to excessive cell growth, proliferation, metastasis, and resistance to apoptosis [60]. Additionally, these mutations alter the HER2 downstream signaling pathway and mutate *PI3K*, *Akt*, and *PDK* genes, contributing to therapeutic resistance [61]. In contrast, *HER2* overexpression is particularly associated with an aggressive tumor phenotype, reduced disease‐free survival (DFS), low overall survival (OS) rate, and poor prognosis [62].

*PIK3CA* is a frequently mutated gene, with mutations identified in approximately 40% of patients with HR‐positive (HR^+^)/HER2^−^ advanced BC patients [63]. These mutations typically arise on chromosome 3q26.3, within a 34 kb *PIK3CA* gene [64]. Functionally, *PIK3CA* regulates several intracellular processes such as cellular transformation, tumor initiation, proliferation, and resistance to cell death [65]. In addition, research has demonstrated that mutations in *PIK3CA* gene can significantly increase PI3Ks catalytic activity, thereby activating the PI3K/Akt signaling pathway and promoting cancer development [66]. Furthermore, somatic mutations in *PIK3CA* confer several advantages to cancer cells, including induced cell growth, stimulated angiogenesis, increased invasion, and resistance to antiestrogen therapy [67]. Clinically, *PIK3CA* mutations are associated with unfavorable outcomes, including decreased OS, reduced relapse‐free survival (RFS), and shorter progression‐free survival (PFS) in BC patients [68].

*BRCA1* and *BRCA2* are tumor suppressor genes that encode enzymes crucial for repairing damaged DNA and are associated with BC pathogenesis [69]. They are involved in transcriptional regulation, cell cycle control, genome stability, the repair of damaged DNA in double‐stranded breaks, and other physiological processes [70]. When *BRCA1* and *BRCA2* undergo loss‐of‐function mutations, the homologous recombination (HR) repair pathway becomes deficient [71]. As a result, these mutations lead to DNA damage, double‐strand breaks, lower fidelity, increased genomic instability, and cancer development [72]. Mutations in *BRCA1* and *BRCA2* genes are reported in approximately 5–10% of BC patients [73]. Moreover, germline mutations in *BRCA1* and *BRCA2* significantly increased the risk of developing BC compared with that in women with wild‐type *BRCA* genes [74]. Individuals carrying these mutations face a significantly higher risk, with an estimated 70% chance of developing BC by the age of 80 years [75].

Partner and localizer of *BRCA2* (*PALB2*) is a tumor suppressor gene located on chromosome 16p12.2 [76]. It plays an essential role in preserving genomic stability, participating in HR repair of DNA double‐strand breaks, and inhibition of cancer progression [77]. Mutations in *PALB2* impair DNA repair mechanisms and significantly increase the risk of cancer development, particularly in BC [78]. Owing to its relatively high mutation frequency and substantial disease penetrance, *PALB2* is now recognized as the third most critical high‐risk BC gene, followed by *BRCA1* and *BRCA2* gene [79]. Carriers of *PALB2* mutations over the age of 40 years face a fivefold to eightfold higher risk of BC, while those under 40 face an eightfold to ninefold increased risk compared with the general population [80]. Clinically, BC patients associated with *PALB2* mutations tend to demonstrate more aggressive pathological characteristics [81]. Moreover, the estimated lifetime risk of BC in women with a *PALB2* mutation is approximately 52.8% by the age of 80 years [82].

The *ESR1* (ER1) gene is located on chromosome 6 at locus 6q24–q27 [83]. It encodes ER alpha (ERα), a key protein or transcription factor (TF) that binds estrogen and regulates several cellular processes, such as cell proliferation, breast tissue development, and the menstrual cycle, and regulates gene expression [48]. Mutations in *ESR1* result in constitutive transcriptional activity and decreased sensitivity to ET [84]. Approximately 30–40% of MBCs treated with ET carry somatic missense mutations in *ESR1* [85]. Mutations in these genes not only contribute to ET resistance but also facilitate metastatic progression [86]. Furthermore, *ESR1* mutations sustain ER activity, altering additional signaling pathways that enable cancer cell proliferation through both ER‐dependent and ER‐independent mechanisms [87]. Clinically, *ESR1* mutations are associated with poor prognosis in MBC patients [88].

The *AT* (ataxia‐telangiectasia) gene is a tumor suppressor gene located on chromosome 11q22.3 [89]. *AT* gene encodes a 350‐kDa protein composed of 3056 amino acids and belongs to the phosphatidylinositol 3‐kinase (PI3K)‐related kinase family [90]. This gene plays a crucial role in genomic integrity, cell cycle regulation, apoptosis, and repair of DNA damage, particularly DNA double‐strand breaks [91, 92]. Molecular alterations in the *AT* gene contribute to carcinogenesis and are commonly observed in approximately 40% of BC patients [93]. The *ATM* gene disrupts cell cycle regulation, apoptosis, oxidative stress, and telomere maintenance, and is an associated risk factor for BC development [93, 94]. Individuals carrying heterozygous *ATM* mutations have a twofold to 13‐fold increased risk of developing BC, especially before the age of 50 years [94]. Recent findings indicate that specific *ATM* variants are linked to an elevated BC risk and poorer prognosis [95].

*TP53* is among the most frequently mutated genes and has been observed in approximately 30–40% of BC patients [96]. Although germline *TP53* mutations are uncommon, occurring in approximately 1% of all BC cases, carriers face a significantly elevated lifetime risk of developing the disease, estimated at 80–90%, which is higher than that associated with most other susceptibility genes [97]. Some *TP53* mutation genes exhibit GoF properties, endowing cancer cells with additional characteristics such as increased proliferative capacity, invasiveness, and metabolic reprogramming, thereby promoting tumor progression [98]. Furthermore, *TP53* encodes the p53 TF, which functions as a tetramer and can interact with homologous proteins TP63 and TP73 [99]. Loss of p53 function leads to critical disruptions in cell cycle control, apoptosis, and DNA repair, thereby promoting tumor growth, proliferation, survival, and resistance to therapy [100].

The *PTEN* gene is a tumor suppressor located on chromosome 10q23 that plays a critical role in regulating the PI3K/Akt signaling pathway [101]. *PTEN* encodes a dual‐specificity phosphatase with both protein and lipid phosphatase activities, acting as a negative regulator of the oncogenic PI3K/Akt cascade [102]. It is involved in essential cellular functions including proliferation, differentiation, migration, adhesion, apoptosis, metastasis, cell cycle control, genome stability, and energy metabolism [103]. Inactivation or mutation of *PTEN* leads to hyperactivation of the PI3K/Akt/mTOR pathway, which promotes uncontrolled growth, cell division, and metastasis [104]. Loss of heterozygosity of *PTEN* has been observed in approximately 40–50% of BC cases, while 5–10% of patients harbor PTEN mutations, primarily frameshift mutations, resulting in loss of PTEN function [105]. Moreover, inactivation or mutation of these genes is linked to more aggressive tumor phenotypes, therapy resistance, and worse clinical outcomes in BC patients [102].

*CDH1* is located on chromosome 16q22.1 and functions as a tumor suppressor gene [106]. It encodes E‐cadherin, a key protein involved in cell–cell adhesion, and transmits intracellular chemical signaling [107]. Mutations in *CDH1* can lead to the loss or dysfunction of E‐cadherin, resulting in abnormal cell proliferation and uncontrolled division [108]. The pathogenic germline variants of *CDH1* are linked to a lifetime risk of up to 55% for invasive lobular carcinoma, primarily due to the downstream loss of E‐cadherin‐mediated adhesion [109]. Furthermore, the reduction or absence of E‐cadherin disrupts the normal tissue architecture, promoting invasion and metastasis, a hallmark of lobular BC [110]. Notably, hereditary lobular BC is strongly associated with germline *CDH1* mutations [111]. Moreover, *CDH1* mutations are involved in hereditary diffuse gastric cancer, increasing the susceptibility to both malignant types in some individuals [112].

**FIGURE 2:**
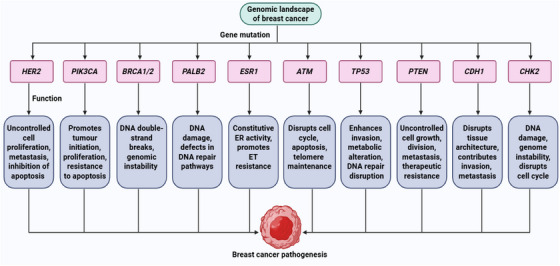
Genomic landscape of BC pathogenesis. First, specific oncogenes and tumor suppressor genes undergo mutations. After that, these mutated genes contribute to several cancer hallmarks. [Created using BioRender.com.]

The *CHEK2* is a tumor suppressor gene located on chromosome 22q12.1 and encodes the serine/threonine kinase CHEK2 [113]. It was initially recognized as the mammalian counterpart of Rad53 in *Saccharomyces cerevisiae* and Cds1 in *Schizosaccharomyces pombe* [114]. *CHK2* plays a vital role in maintaining genomic integrity, participating in the DNA damage response, cell cycle regulation, and apoptosis [115]. However, germline pathogenic variants in *CHEK2* are associated with a moderate elevation in BC risk, by approximately twofold to threefold [73, 116]. Depending on family history, individuals with *CHEK2* mutations may face a lifetime BC risk of 20–44% [117]. Figure 2 depicts the genomic landscape of BC pathogenesis, highlighting the various associated gene mutations.

CNAs are involved in the amplification or deletion of DNA segments [118]. They are located in certain chromosomal regions and are linked to tumor development and progression by affecting oncogenes or tumor suppressor genes [119]. These alterations are common across BC subtypes, with TNBC showing the highest copy number heterogeneity, whereas luminal A/ER^+^ tumors showed the lowest heterogeneous CNA. Frequently, CNAs observed in BC include losses on chromosomes 8p, 13q, 16q, and 17p and gains on chromosomes 1q and 8q [120]. CNAs affect key tumor behaviors, such as proliferation, invasion, metastasis, and resistance to treatment [121]. Certain CNAs are correlated with poor prognosis and increased tumor aggressiveness, making them valuable prognostic markers [122]. Their identification is essential for developing targeted therapies and for advancing personalized cancer diagnostics [123]. Therefore, understanding the specific contributions of CNAs is crucial for developing personalized treatment strategies for BC patients.

### 3.2 TME as Pathogenic Driver

The TME is a complex cellular and molecular landscape that significantly influences cancer progression, including proliferation, angiogenesis, invasion, metastasis, immune evasion, and therapeutic resistance [124]. It consists of tumor cells, immune cells, and various supporting cells such as endothelial cells, stromal cells, and fibroblasts [125]. Moreover, the TME is also involved in the excessive release of bioactive substances, such as chemokines and cytokines [126]. The key cellular and noncellular components of the TME include T cells, regulatory T cells (Treg cells), B cells, endothelial cells, natural killer (NK) cells, tumor‐associated macrophages (TAMs), macrophages, neutrophils, dendritic cells (DCs), myeloid‐derived suppressor cells (MDSCs), cancer‐associated fibroblasts (CAFs), extracellular matrix (ECM), adipocytes, mast cells (MCs), stellate cells, and exosomes [127, 128].

The interaction between malignant and nonmalignant cells within the TME significantly influences cancer initiation and progression [129]. The intricate cellular and molecular communication among TME components supplies nutrients, oxygen, and growth factors that support tumor growth and expansion [130]. Immune cells within the TME exhibit dual roles: while antitumor immune cells eliminate cancer cells during early tumorigenesis, cancer cells counteract this defense by suppressing immune responses through various mechanisms [124]. In the BC microenvironment, infiltrating T cells exhibit elevated PD‐1 expression, whereas antigen‐presenting cells, including DCs, macrophages, and tumor cells, show increased PD‐L1 expression [131]. Notably, PD‐1 expression in CD4^+^ tumor‐infiltrating lymphocytes (TILs) is associated with greater BC invasiveness [132]. In addition, TAMs promote immune escape by releasing interleukin 10 (IL‐10), epidermal growth factor (EGF), and transforming growth factor beta (TGF‐β) [133]. Additionally, MDSCs support tumor progression by inducing angiogenesis, sustaining cancer stem cells (CSCs), and suppressing CD8^+^ T cell activation via nitric oxide synthase 2 and arginase 1 expression [134, 135]. Matrix metalloproteinases (MMPs), particularly MMP‐9 and gelatinase B, facilitate tumor invasion and metastasis by degrading the ECM [136]. In addition, MMP‐9 promotes tumor growth and angiogenesis through the release of VEGF‐A and the suppression of antiangiogenic factors [137]. Importantly, DCs phagocytose apoptotic tumor cells, process tumor antigens, present them via MHC‐I and MHC‐II molecules, migrate to regional lymph nodes, and activate naive CD4^+^ and CD8^+^ T cells to initiate an antitumor immune response [138]. However, tumor cells within the TME actively target DCs to impair their function and suppress the antitumor immune response during BC progression [139]. Research has indicated that tryptase^+^ MCs are involved in tumor progression in both TNBC and luminal A subtype [140]. Furthermore, tumor‐associated stromal cells engage tumor and tumor‐supportive cells to promote cancer progression by secreting protumorigenic cytokines such as IL‐6 and IL‐8 [141]. Moreover, CXCL5 is a key chemokine in the TME and its overexpression is strongly linked to patient survival, recurrence, and metastasis [142].

## 4 Key Signaling Pathways and Therapeutic Targeting

### 4.1 ER Signaling Pathway

ER signaling pathway plays a vital role in regulating cell proliferation, differentiation, and survival in BC [143]. The ER contains conserved domains, including the DNA‐binding and ligand‐binding domain [144]. Its cellular effects are mainly mediated through the nuclear receptors ERα and ERβ, as well as the membrane‐associated G protein‐coupled ER (GPER) [145]. This signaling occurs through two distinct pathways: genomic and nongenomic [48].

**FIGURE 3:**
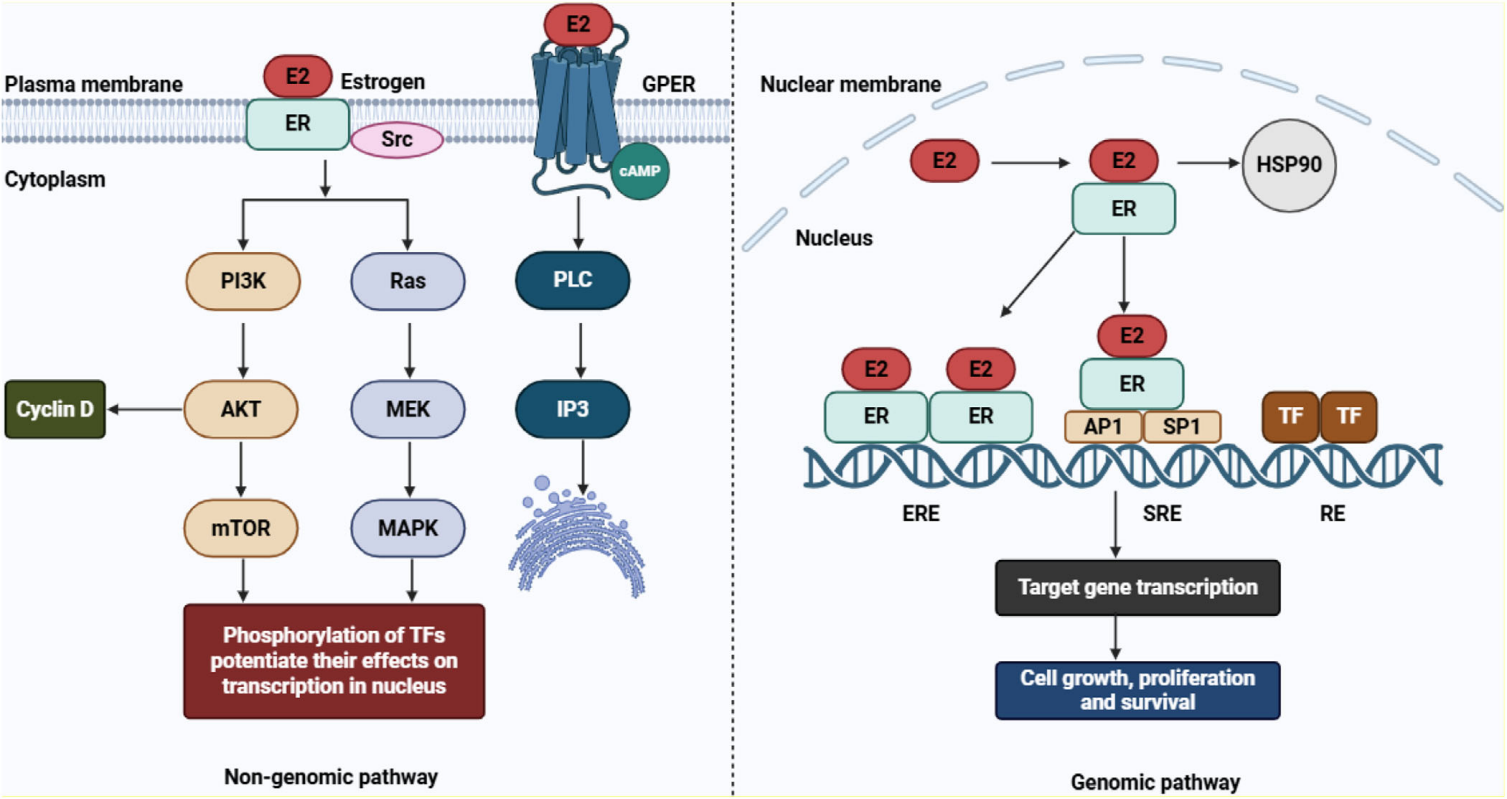
Genomic and nongenomic signaling pathways of estrogen receptor (ER). Akt, serine/threonine kinase; cAMP, cyclic adenosine monophosphate; ERE, estrogen‐response element; GPER, G protein‐coupled estrogen receptor 1; HSP90, heat shock protein 90; IP3, inositol trisphosphate; MAPK, mitogen‐activated protein kinase; MEK, mitogen‐activated extracellular signal‐regulated kinase; mTOR, mammalian target of rapamycin; PI3K, phosphatidylinositide 3‐kinase; PLC, phospholipase C; RE, response element; SRE, serum response elements; TF, transcription factor. [Created using BioRender.com.]

In genomic ER signaling, the estrogen (E2) and ER complex translocates to the nucleus [146]. Upon estrogen binding, ER undergoes a ligand‐specific conformational change that leads to its release from the heat shock protein 90 (HSP90) [147]. In addition, HSP90 acts as a molecular chaperone that stabilizes unbound ER and prevents its degradation [148]. The E2 and ER complex interacts with DNA either directly through estrogen response elements (EREs) or indirectly by associating with other TFs, such as AP1 or SP1, which bind to serum response elements [48]. In indirect action, the complex interacts with DNA‐binding TFs through protein–protein interactions in TF response elements. The ER also regulates TF activity via its association with coactivator proteins [149]. Subsequently, the ER targets specific genes through direct or indirect genomic action. Genes regulated by indirect mechanisms lack EREs in their promoter regions, whereas genes containing EREs are regulated through direct genomic action [150]. In nongenomic ER signaling, estrogen binds to membrane‐associated receptors such as GPER1 or ERs located at the plasma membrane [151]. This pathway is initiated outside the nucleus and functions independent of direct gene transcription. The E2–ER complex primarily triggers intracellular kinase cascades, notably the PI3K/Akt/mTOR and Ras–Raf–MEK–mitogen‐activated protein kinase (MAPK) signaling pathways [152]. These pathways phosphorylate TFs, thereby regulating the transcription of target genes. Figure 3 illustrates the genomic and nongenomic signaling pathways of the ER.

Aberrant expression and signaling of ER contributes to uncontrolled cell growth, proliferation, survival, and drive BC progression [153]. Approximately 70% of BC cases are hormone‐dependent and are classified as luminal A and B subtypes, where tumor cells express ER [154]. Among ER subtypes, ERα plays the most prominent role in BC development [155]. *ESR1*, which encodes ERα, often acquires mutations during the early stages of tumorigenesis [156]. Targeting the ERα signaling pathway offers an effective strategy to control metastasis in BC patients [157]. There are two main types of ET in ER^+^ BC treatment: aromatase inhibitors (AIs) and antiestrogens [158, 159]. AIs, including anastrozole, exemestane, and letrozole, reduce estrogen synthesis [160]. Antiestrogens consist of selective ER modulators (SERMs), such as raloxifene, toremifene, and tamoxifen, which block ERα activation, and selective ER degraders (SERDs), such as fulvestrant, which bind to ERα and promote its degradation, thereby decreasing its activity [161]. These agents inhibit BC growth by disrupting ER signaling [159]. Recently, scientists have examined the combined use of SERM/SERD for the treatment of BC. Mixed SERM/SERDs, including bazedoxifene and lasofoxifene, are being explored as potential therapies for ER^+^ MBC [149, 162].

### 4.2 HER2 Signaling Pathway

The HER2 signaling pathway is essential for the initiation and progression of HER2^+^ BC and represents approximately 15–20% of all BC cases [61]. HER2 is encoded by a gene located on the short arm (q22) of chromosome 17 and is one of the four members of the EGFR family of TKIs [163]. The HER receptor family includes HER1 (EGFR or ErbB1), HER2 (ErbB2), HER3 (ErbB3), and HER4 (ErbB4) [164]. HER2 consists of three main regions: an extracellular ligand‐binding domain, transmembrane domain, and intracellular TK domain [165]. The extracellular domain was further divided into four subdomains (I–IV): [166]. Among these, subdomain IV, located closest to the cell membrane, is crucial for receptor stabilization [167]. In contrast, subdomain II is structurally exposed, making it a key player in heterodimerization with other HER family receptors and facilitating signal transduction [168]. Additionally, the transmembrane domain contains two clusters of cysteine‐rich repeats, which contribute to the structural integrity of receptor [163].

**FIGURE 4:**
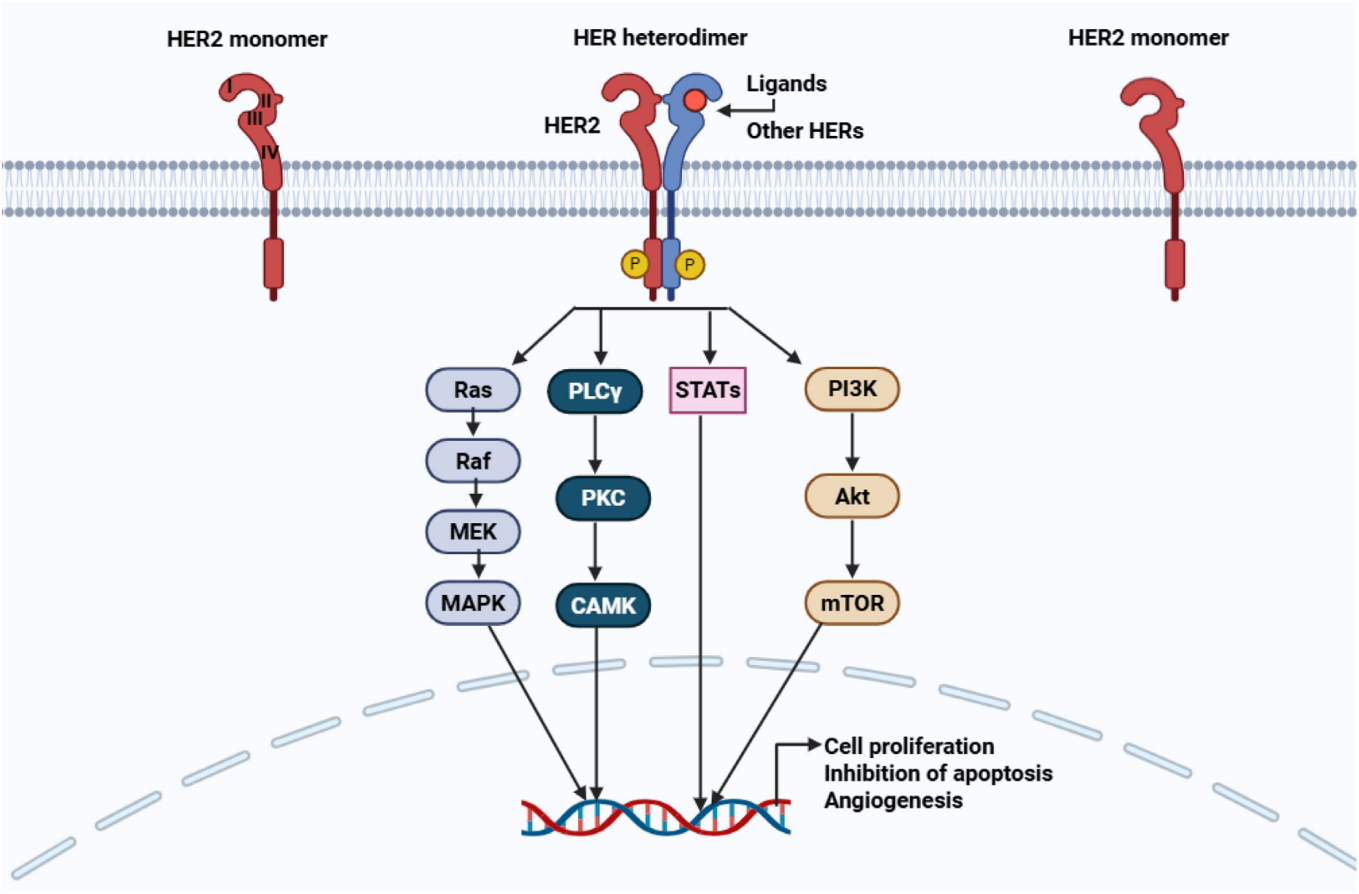
HER2 signaling pathway involvement in BC development. The HER2 signaling activates several downstream signaling cascades, such as the PI3K/Akt/mTOR, Ras/Raf/MEK/MAPK, PKC, and STAT pathways. These pathways contribute to tumor cell proliferation, inhibit apoptosis, and promote metastasis and angiogenesis in BC. Akt, serine/threonine kinase; MAPK, mitogen‐activated protein kinase; MEK, mitogen‐activated extracellular signal‐regulated kinase; mTOR, mammalian target of rapamycin; PI3K, phosphatidylinositide 3‐kinase; PKC, protein kinase C; STAT, signal transducer and activator of transcription. [Created using BioRender.com.]

HER2 is distinct from other members of the EGFR family owing to its inability to form ligand‐dependent homodimers. Instead of functioning independently, it requires heterodimerization with other receptors from the EGFR family or can spontaneously undergo homodimerization when overexpressed [169]. In contrast, HER1, HER3, and HER4 are activated through ligand binding, which induces conformational changes that allow dimerization, either with each other or with HER2, leading to activation of their intracellular TK domains [61]. Following dimerization, receptors undergo autophosphorylation and trans‐phosphorylation at specific tyrosine residues, triggering a cascade of downstream signaling events [170]. These phosphorylation sites serve as docking points for adaptor proteins that activate several key signaling pathways, including the PI3K/AKT/mTOR, MAPK, protein kinase C (PKC), and STAT pathways [171, 172]. These signaling cascades collectively modulate critical cellular processes by altering gene expression through TF activation, transcript modification, and regulation of cell cycle proteins [149, 173]. Ultimately, this drives malignant transformation by promoting tumor cell proliferation, survival, resistance to apoptosis, metastasis, and angiogenesis [174, 175]. Figure 4 highlights the HER2 signaling cascade pathway and its involvement in BC development.

### 4.3 PI3K/Akt/mTOR Pathway

The PI3K/Akt/mTOR signaling pathway is essential for regulating fundamental cellular processes such as cell growth, differentiation, proliferation, cell cycle regulation, apoptosis, metastasis, and angiogenesis [176, 177]. This pathway involves multiple key molecules including PI3K, Akt, mTOR, receptor TKs (RTKs), G protein‐coupled receptors (GPCRs), and phosphoinositide‐dependent protein kinase 1 (PDK1) [178].

PI3Ks are intracellular lipid kinases that serve as key mediators in signal transduction by relaying signals from transmembrane receptors such as RTKs and GPCRs to regulate a variety of cellular functions [179]. The PI3K family is divided into three distinct classes: Class I, Class II, and Class III [180]. Class I PI3Ks possess a heterodimeric structure comprising of both catalytic and regulatory subunits. The catalytic subunits of Class IA PI3Ks include p110α, p110β, p110γ, and p110δ, which are encoded by *PIK3CA*, *PIK3CB*, *PIK3CG*, and *PIK3CD*, respectively, [181]. Meanwhile, regulatory subunits, such as p85α (with isoforms p85α, p55α, and p50α), p85β, and p55γ, are encoded by the genes *PIK3R1*, *PIK3R2*, and *PIK3R3* [182]. Each catalytic subunit within the PI3K family possesses the intrinsic capability to associate with its respective regulatory subunit, thereby modulating downstream signaling pathways [183]. Class IB PI3Ks contain the catalytic subunit p110γ, regulatory subunit p101, and p84/p87PIKAP subunits [179]. In contrast to Class I, Class II PI3Ks are monomeric proteins with high molecular weights and do not require regulatory subunits [184, 185]. Class II PI3Ks are represented by three isoforms, including PIK3C2α, PIK3C2β, and PIK3C2γ, which have distinct roles in regulating intracellular membrane dynamics, vesicular trafficking, and other biological processes [179, 186]. Class III PI3Ks are heterodimers, comprising the catalytic subunit hVps34 and the regulatory subunit p150, which collectively govern fundamental cellular activities, including endosomal sorting, pinocytosis, phagocytosis, and autophagy [187, 188].

**FIGURE 5:**
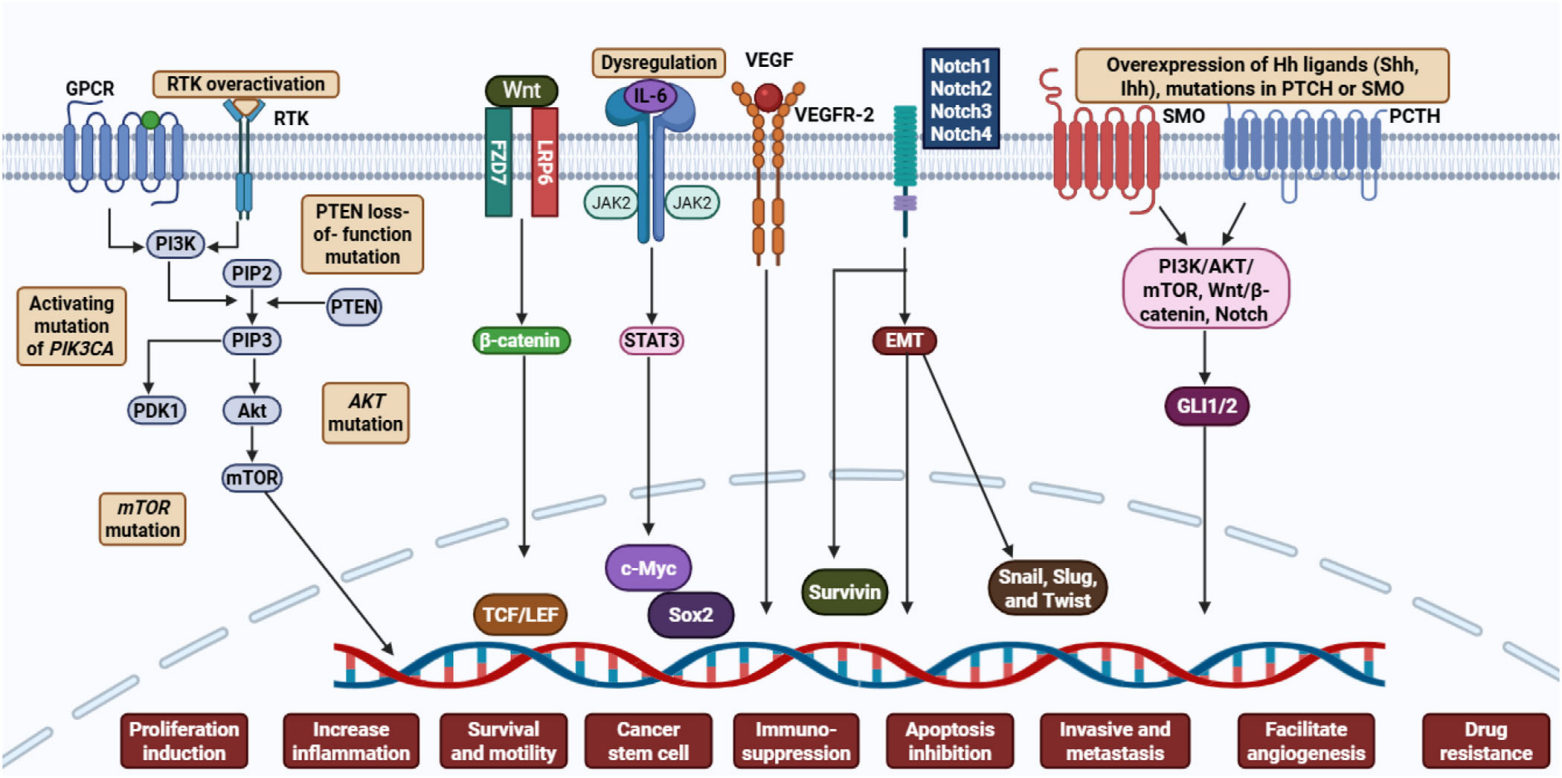
Various cell signaling pathways involvement in BC progression. The dysregulation of different signaling cascades, such as PI3K/Akt/mTOR, Wnt/β‐catenin, JAK2/STAT3, Notch, and Hedgehog contributes to tumor cell proliferation, survival, CSC maintenance, immunosuppression, apoptosis inhibition, and the enhancement of metastasis and angiogenesis in BC. Akt, serine/threonine kinase; BC, breast cancer; CSC, cancer stem cell; JAK2, Janus kinase 2; mTOR, mammalian target of rapamycin; PI3K, phosphatidylinositide 3‐kinase; STAT3, signal transducer and activator of transcription‐3. [Created using BioRender.com.]

The activation of PI3K requires the binding of growth factors to RTKs such as insulin receptor, insulin‐like growth factor 1 receptor, and HER [189]. Additionally, PI3K activation can occur through GPCRs and oncogenic signaling, particularly via Ras [190, 191]. Upon activation, Class I PI3K catalyzes the phosphorylation of phosphatidylinositol 4,5‐bisphosphate (PIP2), leading to the production of PIP3 [181]. The generation of PIP3 triggers downstream activation of various kinases, including PDK1 and Akt [192]. In addition, Akt is the primary molecule of the PI3K pathway and exists in three isoforms: Akt1, Akt2, and Akt3 [193]. Upon activation, Akt translocates to the cell membrane, where it undergoes phosphorylation at Ser473 by mTORC2 and Thr308 by PDK1 [194]. Activated Akt phosphorylates a variety of target proteins across the cell membrane, cytosol, and nucleus [195]. These phosphorylated targets trigger crucial cellular responses, including the stimulation of cell proliferation, division, and survival [196]. Additionally, activated Akt induces the phosphorylation of mTOR, which exists in two separate multiprotein complexes, mTOR complex 1 (mTORC1) and mTORC2 [197]. These complexes regulate various cellular processes, including the modulation of Akt phosphorylation and activation [198]. PTEN functions as a negative regulator of this pathway by dephosphorylating PIP3 to PIP2 [199]. Since PIP3 serves as a docking site for Akt activation, its dephosphorylation by PTEN prevents Akt activation, thereby suppressing the PI3K/Akt/mTOR signaling cascade [200]. In BC progression, dysregulation of the PI3K/Akt/mTOR pathway frequently arises due to genetic alterations, including mutations in *PIK3CA*, *Akt*, and *mTOR*, as well as overexpression of RTKs or loss of PTEN function [201]. These molecular aberrations contribute to uncontrolled cell proliferation, survival, metastasis, angiogenesis, and resistance to therapy, making this pathway a crucial target for the management of BC [42, 202]. Figure 5 illustrates the involvement of various cell‐signaling pathways in BC progression.

### 4.4 Wnt/β‐Catenin Pathway

The canonical Wnt/β‐catenin signaling pathway, often referred to as the Wnt/β‐catenin cascade, is crucial for various physiological processes, such as embryogenesis, stem cell regulation, oncogenesis, and the development of resistance to therapeutic agents [203, 204]. Dysregulation of this pathway is commonly observed in BC, where it plays a key role in increasing tumor aggressiveness, metastatic progression, and unfavorable clinical outcomes [205, 206]. Within this signaling cascade, specific Wnt ligands, such as Wnt1, Wnt3a, and Wnt10b, interact with Frizzled receptors and low‐density lipoprotein receptor‐related proteins, thereby disrupting the function of the β‐catenin destruction complex, which consists of APC, Axin, CK1, and GSK‐3β [207, 208]. By inhibiting the degradation of β‐catenin, this pathway enables its cytoplasmic accumulation, followed by its translocation into the nucleus, where it engages with TCF/LEF TFs to induce the activation of certain genes associated with uncontrolled cellular proliferation, stemness, and survival, thereby significantly assisting BC progression [209, 210]. This pathway has been implicated in promoting epithelial‐to‐mesenchymal transition (EMT), which enhances cancer cell invasiveness and metastatic potential by upregulating key EMT markers including Slug, Snail, ZEB1, and Twist [211, 212].

Aberrant activation of the Wnt/β‐catenin signaling pathway is particularly prominent in TNBC, a highly aggressive BC subtype characterized by therapy resistance and a high rate of recurrence [213]. In HER2^+^ BC, the interaction between the Wnt signaling cascade and HER2/PI3K/Akt pathways synergistically accelerates tumor progression [26, 214]. Additionally, in ER^+^ BC, activation of Wnt signaling plays a crucial role in driving resistance to ET, such as tamoxifen, thereby diminishing therapeutic efficacy [215]. A recent study reported a novel mechanism underlying β‐catenin activation in TNBC, including upregulation of Src homology and collagen 4, which facilitates Src kinase autophosphorylation and, in turn, modulates the β‐catenin pathway to drive TNBC metastasis [216].

### 4.5 JAK2/STAT3 Signaling Pathway

The JAK2/STAT3 signaling pathway consists of JAK2, a non‐RTK, and STAT3 proteins [217]. JAK2 plays a role in multiple signaling mechanisms, whereas STAT3 is essential for controlling cell growth, differentiation, and apoptosis by translocating to the nucleus [218, 219]. This pathway is pivotal for the formation of inflammatory microenvironments that promote cancer development [220].

In BC, dysregulated activation of the JAK2/STAT3 signaling cascade promotes unregulated tumor proliferation, facilitates angiogenesis, enhances metastasis, and induces resistance to therapeutic agents [221]. This aberrant signaling is particularly exhibited in TNBC, where hyperactivation is linked to an aggressive tumor nature and unfavorable clinical outcomes [222]. In HER2^+^ BC, JAK2/STAT3 is intricately linked to the HER2/PI3K/Akt axis, contributing to the development of resistance to HER2‐targeted therapeutics [223]. Likewise, in ER^+^ BC, IL‐6‐induced activation of JAK2/STAT3 has been associated with the emergence of resistance to endocrine therapies, further complicating patient treatment outcomes [224]. This activation promotes the upregulation of cyclin D1 and c‐Myc, which drives increased cell proliferation, growth, and survival [225, 226]. Meanwhile, antiapoptotic proteins such as Bcl‐XL and Mcl‐1 enhance cell survival and contribute to chemotherapy resistance [227, 228]. Notably, JAK2/STAT3 signaling supports CSC self‐renewal, which is crucial for tumor recurrence and resistance to treatment [229]. This pathway also promotes EMT by upregulating Twist, Slug, and Snail, thereby enhancing the invasion and metastatic potential of BC cells [230]. Moreover, STAT3‐mediated upregulation of VEGF enhances angiogenesis by stimulating its receptor, VEGFR‐2 [222, 231]. This process not only increases the delivery of oxygen and nutrients to the growing tumor cells but also promotes the expression of c‐Myc and Sox2, which are essential for sustaining tumor growth and progression [232].

### 4.6 Notch Signaling Pathway

The Notch signaling pathway is a highly conserved and intricate cellular communication system that is pivotal in regulating tumorigenesis and cancer progression, including cell growth, differentiation, and self‐renewal [233]. This pathway also exerts a regulatory role in stem cell maintenance and facilitates EMT, thereby enhancing the metastatic potential of malignant cells [234]. Structurally, this pathway comprises a family of Notch receptors (Notch1–4) and their corresponding ligands, which belong to two distinct subclasses: jagged (Jag1, Jag2) and Delta‐like (Dll1, Dll3, Dll4) [235]. The Notch signaling pathway is activated when a ligand interacts with the Notch receptor, inducing a proteolytic cleavage event mediated by γ‐secretase [234]. This cleavage releases the Notch intracellular domain (NICD), which is subsequently translocated to the nucleus [236]. Once inside the nucleus, NICD interacts with CSL TFs (CBF1/Su(H)/Lag‐1), facilitating the transcription of target genes that govern cell fate determination, growth, and proliferation [237].

Notch signaling is essential for regulating mammary gland development and tissue homeostasis [238]. However, its dysregulation can play a major role in the initiation of BC, with aberrant activation frequently serving as an early and critical event in tumor progression [239]. Such dysregulation promotes various hallmarks of cancer, including tumor cell proliferation, metastasis, EMT, and CSCs properties [240, 241]. Notably, overexpression of Notch1 and Notch4 has been strongly associated with TNBC, where it drives the self‐renewal of CSCs and increases their resistance to therapeutic agents [242]. Notch activation further facilitates tumor progression by promoting angiogenesis through upregulation of VEGF expression [12]. It also contributes to radioresistance by enhancing DNA repair mechanisms [243]. Moreover, dysregulated Notch signaling intricately crosstalk with other oncogenic pathways, including PI3K/Akt and Wnt/β‐catenin, establishing a potential connection between protumorigenic networks that amplify malignancy and therapy resistance [244].

### 4.7 Hedgehog (Hh) Signaling Pathway

The Hedgehog (Hh) signaling pathway is an essential mediator of embryonic development and cellular differentiation [245]. This pathway consists of three primary ligands: Sonic Hh (Shh), Desert Hh, and Indian Hh (Ihh), along with the transmembrane receptor Patched (PTCH) and GPCR Smoothened (SMO) [246]. Under physiological conditions, PTCH1 inhibits SMO, thereby maintaining its inactive state [247]. However, upon binding of Hh ligands to PTCH1, this inhibitory effect is relieved, enabling SMO to activate downstream effectors, including GLI1, GLI2, and GLI3 [248]. These TFs regulate the activation of genes that govern cellular proliferation, survival, and the maintenance of stemness [249].

In BC, abnormal activation of the Hh signaling cascade enhances tumor growth and resistance to therapy [41]. This activation can result from the overexpression of Hh ligands such as Shh and Ihh, mutations in PTCH1 or SMO, and epigenetic modifications [250]. This pathway is essential for enhancing CSCs self‐renewal, increasing drug resistance, and facilitating metastasis by inducing the EMT [212]. Additionally, it engages with other signaling pathways, such as Wnt/β‐catenin and Notch, further driving the progression and development of BC [251]. Similar to the Notch pathway, the Hh pathway is significantly active in TNBC, playing a key role in its aggressiveness and unfavorable prognosis [252]. Increased expression of the Shh ligand has been reported to be correlated with lower survival rates in TNBC [253]. Furthermore, GLI1 and GLI2 exhibit higher basal expression levels in TNBC than in HR^+^ BC [254]. Knocking out GLI1 decreases BC cell viability, whereas its elevated expression in HR^−^ BC is linked to poor prognosis [255, 256]. In TNBC, the Hh pathway drives the stemness of cancer cells, activates CAFs, enhances the invasive capacity of BC, and promotes angiogenesis by upregulating GLI1 and GLI2 TFs [216].

### 4.8 Cell Cycle Dysregulation

The cell cycle consists of a series of highly regulated phases that guide cell growth, DNA replication, and division in an acute manner [257]. Key components in this regulation include cyclins, CDKs, CDK inhibitors, and retinoblastoma protein (Rb), all of which coordinate the transitions between different phases [258]. Disruption of this regulation is a hallmark of cancer and leads to uncontrolled cell growth and cancer progression [259].

**FIGURE 6:**
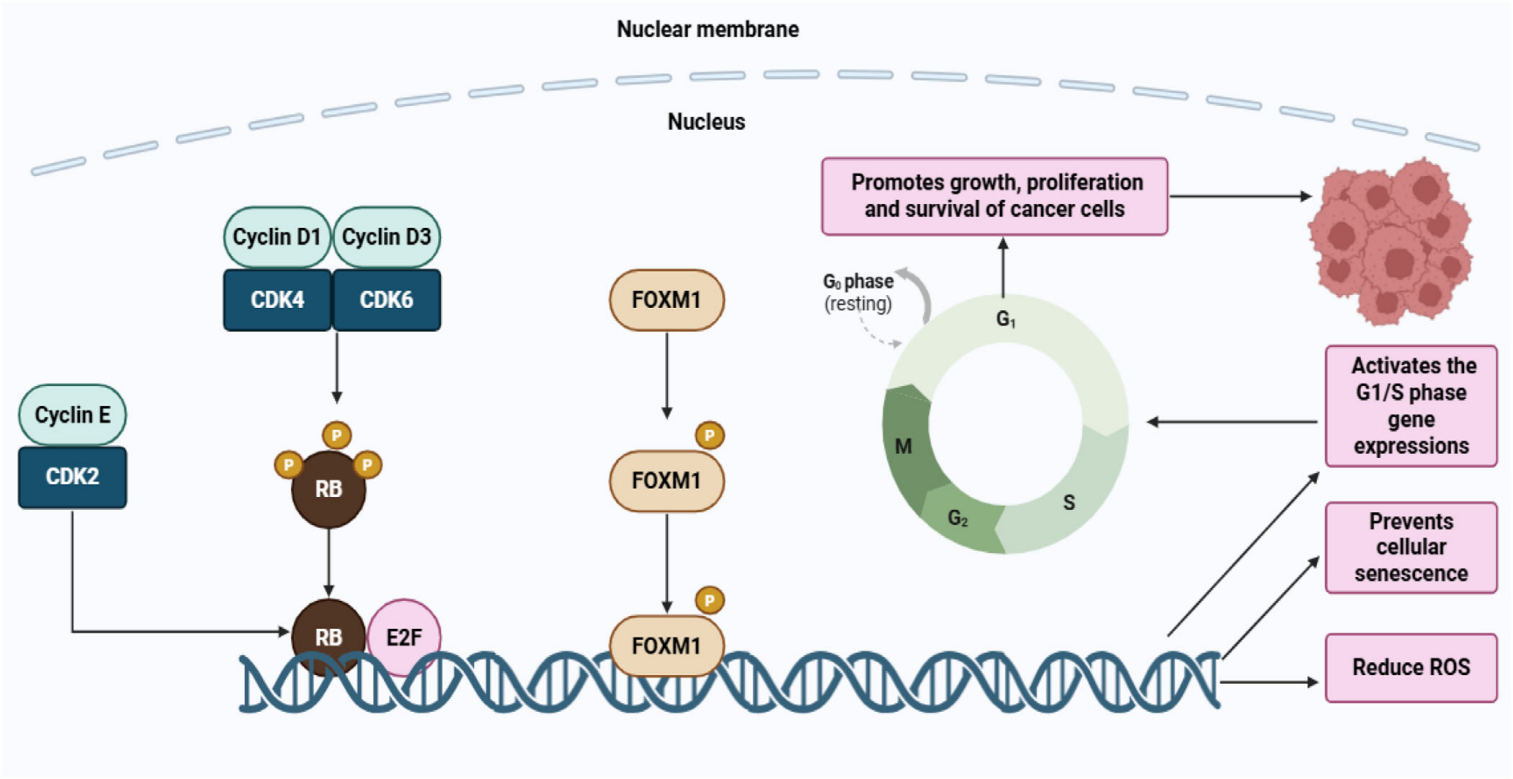
Cell cycle dysregulation in BC. CDK4/6 leads to Rb activation through its phosphorylation. Similarly, cyclin E in complex with CDK2 contributes to Rb phosphorylation. Once activated, Rb facilitates cell cycle progression by activating E2F transcription factors. In addition, CDK4 and CDK6 promote the phosphorylation‐dependent stabilization and activation of FOXM1, subsequently reducing ROS, prevents cellular senescence, and enhances the expression of G1/S phase genes, thereby promoting cancer cell growth, proliferation, and survival. BC, breast cancer; CDK: cyclin‐dependent kinase; E2F, early region 2 binding factor; FOXM1, forkhead Box M1; ROS, reactive oxygen species. [Created using BioRender.com.]

CDKs are essential for the regulation of cell division by controlling cell cycle progression [260]. One key player in this regulation is the Rb protein, a tumor suppressor that prevents the transition from G1 phase to S phase [261]. This is achieved by binding to E2F TFs, resulting in the blockage of the early stages of cell division [262]. During the G1 phase, CDK4 and CDK6 form complexes with cyclin D1, D2, and D3 in response to various growth signals, facilitating cell cycle [263]. The CDK4/6‐cyclin D complex phosphorylates Rb, leading to the release of the E2F TFs [264]. This release facilitates the cell to proceed from the G1 phase to the S phase, promoting cell cycle progression [265]. CDK4 and CDK6 are important for the growth and differentiation of cancer cell [266]. Their activation by D‐type cyclins, such as cyclin D1/CDK4 and cyclin D3/CDK6, drives the phosphorylation of Rb, further supporting uncontrolled division of cancerous cells [267]. Phosphorylated Rb (phospho‐Rb) facilitates cell cycle progression and division by activating E2F transcription [268]. In BC, abnormal cell cycle progression is commonly driven by cyclin E overexpression, CDK4/6 amplification, and loss‐of‐function mutations in tumor suppressor genes like p53 [269]. Similarly, in HR^+^ BC, cyclin D is often upregulated, leading to the restoration of phospho‐Rb expression [270]. Therefore, the G1/S checkpoint is a critical therapeutic target for halting BC cell growth and proliferation [271]. Forkhead box M1 (FOXM1) is a key TF and crucial phosphorylation target of CDK4/6 [272, 273]. These kinases promote the stabilization and activation of FOXM1 through phosphorylation, which in turn enhances the expression of genes involved in the G1 to S phase transition [274]. Moreover, FOXM1 activation reduces reactive oxygen species levels, prevents cellular senescence, and promotes survival, growth, and proliferation of cancer cells [275]. Figure 6 illustrates the cell cycle dysregulation in BC.

## 5 Molecular Subtype‐Specific Targeted Therapies

### 5.1 HR^+^/HER2^−^ BC

#### 5.1.1 CDK4/6 Inhibitors

CDK4/6 inhibitors have shown promise as therapeutic agents for HR^+^/HER2^−^ BC [276]. They specifically inhibit CDK4/6 activity and prevent phosphorylation of Rb protein during the G1 phase, thereby halting the transition from G1 to S phase [277]. The United States Food and Drug Administration (US FDA) has approved three major CDK4/6 inhibitors, palbociclib, ribociclib, and abemaciclib, to be used in combination with ET to treat HR^+^/HER2^−^ BC [278]. These inhibitors have revolutionized MBC by directly targeting the cell cycle machinery [279]. They disrupt intracellular and mitogenic hormone signaling pathways that drive the uncontrolled proliferation of cancerous cells [280].

Palbociclib is the first orally active and selective CDK4/6 inhibitor approved for the treatment of BC [281]. In 2015, the US FDA approved palbociclib in combination with letrozole for patients with disease progression, despite prior ET [282]. The following year, the US FDA granted full approval for its use alongside fulvestrant in women with HR^+^, HER2^−^ MBC whose disease had worsened after endocrine treatment [283]. By 2017, palbociclib was further approved as a first‐line therapy in combination with an AI for postmenopausal women [284]. It has been shown to be effective in both premenopausal and postmenopausal women with HR^+^/HER2^−^ BC [285]. However, it may lead to several side effects, including an increased risk of diarrhea, back pain, neutropenia, and pulmonary embolism [286, 287]. Ribociclib is a highly selective and orally bioavailable CDK4/6 inhibitor used in the therapeutic management of HR^+^ and HER2^−^ BC [288]. Its clinical use is characterized by a relatively favorable toxicity profile, making it a viable treatment option in clinical practice [289]. Ribociclib is commonly administered in combination with hormone‐blocking agents to maximize its efficacy in suppressing tumor growth and progression [290]. In 2017, the US FDA approved ribociclib as a first‐line treatment in combination with an AI such as letrozole for postmenopausal women with HR^+^/HER2^−^ MBC [291]. Although ribociclib is generally well tolerated, its use is associated with several adverse effects, including nausea, vomiting, diarrhea, fatigue, neutropenia, and leukopenia [292, 293]. Abemaciclib demonstrated a more potent inhibitory effect on CDK4/6 enzymes in vitro than other CDK4/6 inhibitors [294]. It has also shown inhibitory activity against other kinases, such as CDK9 and the proto‐oncogene serine/threonine–protein kinase (PIM1), which may contribute to its broader therapeutic potential [295]. In 2017, the US FDA approved abemaciclib for the treatment of postmenopausal women with HR^+^/HER2^−^ advanced BC or MBC [296]. It has received approval for use along with ET [297]. Compared with other CDK4/6 inhibitors, abemaciclib has been noted to have less frequent hematopoietic toxicity [298]. As a result, it has a lower incidence of neutropenia, but is linked to an increased rate of diarrhea and an elevated risk of severe lung inflammation [299, 300]. Table 2 displays the clinical trials that assessed the use of CDK4/6 inhibitors in the management of HR^+^/HER2^−^ BC.

**TABLE 2:** Summary of clinical trials assessing CDK4/6 inhibitors for HR^+^/HER2^−^ BC management.

CDK 4/6 inhibitor	Population	Treatment	NCT ID	Period	Phases	Status
Palbociclib	HR^+^/HER2^−^ MBC or local ABC	ET	NCT04546009	09‐10‐2020 to 25‐03‐2028	III	Active, not recruiting
	HR^+^/HER2^−^ MBC, treatment naive	ET	NCT03870919	23‐10‐2019 to 27‐10‐23	N/A	Active, not recruiting
	HR^+^/HER2^−^ isolated locoregional recurrent BC	ET	NCT03820830	27‐08‐2019 to 01‐01‐2029	III	Active, not recruiting
	HR^+^/HER^−^ ABC	ET	NCT03423199	09‐02‐2018 to 09‐2025	III	Active, not recruiting
	HR^+^/HER2^−^ ABC or MBC	ET	NCT03332797	24‐11‐2017 to 30‐06‐2026	I	Active, not recruiting
	HR^+^ early‐stage BC	ET	NCT02764541	24‐05‐2016 to 04‐2031	II	Active, not recruiting
Ribociclib	HR^+^/HER2^−^ early BC	ET	NCT03701334	07‐12‐2018 to 29‐05‐2026	III	Active, not recruiting
	HR^+^/HER2^−^ ABC	ET	NCT03944434	27‐12‐2018 to 27‐11‐2025	II	Active, not recruiting
	HR^+^/HER2^−^ ABC	ET	NCT03671330	29‐08‐2018 to 09‐05‐2025	II	Completed
Abemaciclib	HR^+^/HER2^−^ MBC	ET	NCT04158362	11‐06‐2020 to 06‐2028	III	Active, not recruiting
	HR^+^/HER2^−^ early BC	ET	NCT04293393	02‐10‐2020 to 28‐02‐2033	II	Active, not recruiting
	HR^+^/HER2^−^ ABC	ET	NCT04352777	14‐09‐2020 to 25‐06‐2025	II	Completed
Dalpiciclib	HR^+^/HER2^−^ early BC	ET	NCT06341894	17‐11‐2023 to 06‐2029	III	Recruiting

*Data sources*: ClinicalTrials.gov.

Multiple clinical trials, including PALOMA, MONALEESA, and DAWNA, have investigated the safety and efficacy of ET with CDK4/6 inhibitors in the treatment of HR+/HER2^−^ BC [301]. In addition, PALOMA‐1, PALOMA‐2, MONARCH‐3, MONALEESA‐2, and MONALEESA‐7 have investigated the use of CDK4/6 inhibitors in combination with ET as first‐line treatment for HR^+^/HER2^−^ BC, showing a notable improvement in PFS compared with ET alone [302]. Additionally, the MONALEESA‐7 trial, which was conducted in premenopausal women with HR^+^/HER2^−^ MBC, demonstrated notable enhancements in PFS and OS [303]. Unlike palbociclib and ribociclib, abemaciclib is approved for use as a monotherapy owing to its distinct off‐target activity [304]. These findings have established CDK4/6 inhibitors plus ET as the standard treatment for HR^+^/HER2^−^ MBC [305]. Furthermore, the PALOMA‐3, MONARCH‐2, and MONALEESA‐3 trials evaluated the combination of CDK4/6 inhibitors and ET in patients resistant to prior endocrine treatment [306]. Compared with endocrine monotherapy, these studies reported significant increases in the median PFS (mPFS) and objective response rates (ORR) [307]. Although MONARCH‐2 and MONALEESA‐3 demonstrated a statistically significant improvement in OS, PALOMA‐3 showed a statistically nonsignificant trend toward OS enhancement [308]. Notably, dalpiciclib has recently received approval from the China FDA for the treatment of HR^+^/HER2^−^ MBC [309]. It has also been shown to overcome resistance to trastuzumab and tamoxifen in BC [310]. The DAWNA‐1 phase trial evaluated dalpiciclib, with or without fulvestrant, in HR^+^/HER2^−^ MBC patients previously treated with ET and demonstrated significantly improved PFS [311]. In the DAWNA‐2 trial, dalpiciclib combined with ET as a first‐line treatment resulted in a mPFS of over 30 months and a 49% reduction in disease progression risk [306]. Moreover, tibiremciclib (BPI‐16350), a novel CDK4/6 inhibitor, has shown effectiveness as a second‐line treatment for HR^+^/HER2^−^ BC [312]. When combined with fulvestrant, it significantly improved PFS compared with placebo plus fulvestrant in patients with HR^+^/HER2^−^ advanced BC patients [313]. Table 3 summarizes CDK4/6 inhibitors and survival outcomes of patients with HR^+^/HER2^−^ BC.

**TABLE 3:** CDK4/6 inhibitors and survival outcome for HR^+^/HER2^−^ BC patients.

Name of clinical trail	Patients	Treatment	*N*	PFS (month)	OS	References
PALOMA‐1	Postmenopausal women with HR^+^/HER2^−^ ABC or MBC	Palbociclib + letrozole vs. letrozole	165	20.2 vs. 10.2	Nonsignificant	[314]
PALOMA‐2	Postmenopausal women with HR^+^/HER2^−^ ABC or MBC	Palbociclib + letrozole vs. letrozole	666	24.8 vs. 14.5	53.9 vs. 51.2	[315]
PALOMA‐3	Pre and postmenopausal women with HR^+^/HER2^−^ ABC or MBC	Palbociclib + fulvestrant vs. placebo + fulvestrant	521	9.5 vs. 4.6	34.8 vs. 28.0	[316]
MONARCH‐2	Pre and postmenopausal women with HR^+^/HER2^−^ ABC or MBC	Abemaciclib + fulvestrant vs. placebo + fulvestrant	669	16.4 vs. 9.3	46.7 vs. 37.3	[317]
MONARCH‐3	Postmenopausal women with HR^+^/HER2^−^ ABC or MBC	Abemaciclib + ET vs. placebo + ET	493	28.1 vs. 14.7	66.8 vs. 53.7	[318]
MONALEESA‐2	Postmenopausal women with HR^+^/HER2^−^ ABC or MBC	Ribociclib + letrozole vs. letrozole + placebo	668	25.3 vs. 16	63.9 vs. 51.4	[319]
MONALEESA‐3	Postmenopausal women with HR^+^/HER2^−^ ABC or MBC	Ribociclib + letrozole vs. placebo + letrozole	726	20.5 vs. 12.8	53.7 vs. 41.5	[320]
MONALEESA‐7	Pre or postmenopausal women with HR^+^/HER2^−^ ABC or MBC	Ribociclib + ET vs. placebo + ET	672	23.8 vs. 13.0	58.7 vs. 48.0	[321]
DAWNA‐1	Pre or postmenopausal women with HR^+^/HER2^−^ ABC or MBC	Dalpiciclib + fulvestrant vs. fulvestrant + placebo	361	15.7 vs. 7.2	—	[322]
DAWNA‐2	Pre or postmenopausal women with HR^+^/HER2^−^ ABC or MBC	Dalpiciclib + letrozole vs. letrozole + placebo	456	30.6 vs. 18.2	—	[323]

Despite notable advancements in improving patient outcomes in HR^+^/HER2^−^ BC using CDK4/6 inhibitors, these therapies are associated with several side effects, including neutropenia, cardiac issues, pneumonitis, diarrhea, and elevated levels of alanine aminotransferase (ALT) and/or aspartate aminotransferase [309, 324]. Additionally, CDK4/6 inhibitors remain susceptible to both primary and secondary resistance [325]. Notably, acquired *RB1* mutations have been identified as a mechanism that contributes to resistance to CDK4/6 inhibitor therapy [326]. Additional mechanisms of resistance to CDK4/6 inhibitors include amplification of CCN1 or CDK4/6, loss‐of‐function mutations in *TP53*, and overexpression of both the cyclin E–CDK2 complex and E2F [271, 327]. Other reported resistance pathways involved alterations in Akt1, CCNE, ERBB2, FGFR2, RAS, and AURKA [328]. Notably, it has been demonstrated that activation of the PI3K/Akt/mTOR signaling pathway is associated with CDK4/6 inhibitor resistance [329]. Consequently, targeting this pathway with PI3K or mTOR inhibitors represents a potential therapeutic strategy for HR^+^/HER2^−^ BC patients with CDK4/6 inhibitor resistance [330].

#### 5.1.2 PI3K/Akt/mTOR Inhibitors

##### 5.1.2.1 Alpelisib

Alpelisib is the first orally administered PI3K inhibitor specifically engineered to selectively target the p110α isoform of PI3K enzyme [331]. In 2019, the US FDA approved alpelisib for use in conjunction with the ER antagonist fulvestrant [332]. This combination is intended for postmenopausal women and men diagnosed with HR^+^/HER2^−^, *PIK3CA*‐mutated advanced or MBC [333]. It is recommended for patients whose condition worsens during or after endocrine treatment [334]. In both preclinical and early clinical trials, a combination of alpelisib and fulvestrant, a SERD, showed significant anticancer activity [335]. In addition, mPFS corresponding to a 35% reduction in the risk of disease progression or death was notably extended to 11 months with alpelisib and fulvestrant combination compared with 5.7 months with placebo and fulvestrant [332]. Additionally, the median OS (mOS) was 39.3 months in the alpelisib group versus 31.4 months in the placebo group. Based on these promising results, the phase III SOLAR‐1 trial was designed to evaluate the effectiveness of this combination in patients with HR+/HER2^−^ BC carrying *PIK3CA* mutations [336]. The most commonly reported adverse outcomes in patients treated with alpelisib include nausea, diarrhea, fatigue, decreased appetite, hyperglycemia,  stomatitis, rash, and gastrointestinal disturbances [337, 338].

##### 5.1.2.2 Inavolisib

Inavolisib (GDC‐0077) is a PI3Kα‐selective inhibitor that has shown promising antitumor activity in patients with *PIK3CA*‐mutated HR^+^/HER2^−^ locally advanced BC (LABC) or MBC [339]. Biochemical studies have revealed that inavolisib is more than 300‐fold selective for p110α compared with p110β, p110δ, and p110γ isoforms [340]. It also exhibits greater potency in tumor cells harboring mutated p110α than in those harboring wild‐type p110α, both as a monotherapy and in combination with ET [341]. The US FDA approved inavolisib on October 10, 2024, in combination with palbociclib and fulvestrant for the treatment of adults with endocrine‐resistance, *PIK3CA*‐mutated HR^+^/HER2^−^ LABC, or MBC [342]. The NAVO120 trial evaluated the inavolisib combination with palbociclib and fulvestrant versus placebo with palbociclib and fulvestrant as first‐line treatment in patients who relapsed during or within 12 months following adjuvant ET [343]. Preliminary findings indicated a significantly positive result, with a median follow‐up of 21.3 months and a longer mPFS of 15.0 months compared with 7.3 months in the placebo group [344]. Moreover, INAVO121 is an ongoing phase III study assessing the efficacy and safety of inavolisib with fulvestrant versus alpelisib with fulvestrant following progression to CDK4/6 inhibitors [345]. The most frequently reported adverse events in the inavolisib group included diarrhea, nausea, headache, rash, anemia, decreased appetite, neutropenia, thrombocytopenia, leukopenia, hyperglycemia, and stomatitis [346].

##### 5.1.2.3 Taselisib

Taselisib (GDC‐0032) is an oral PI3Kα inhibitor that specifically targets α, δ, and γ isoforms of the PI3K pathway [347]. Unlike pan‐PI3K inhibitors, taselisib is believed to have fewer side effects and greater efficacy in treating *PIK3CA*‐mutant cancers, owing to its selective action on these specific PI3K isoforms [348]. In May 2019, the US FDA approved taselisib as the first PI3K inhibitor to be used against HR^+^/HER2^−^ BC with a *PIK3CA* mutation [349]. The combination of taselisib, palbociclib, and fulvestrant was evaluated in patients, heavily pretreated with HR^+^ BC carrying *PIK3CA*‐mutant, with a therapy response rate in *PIK3CA‐*mutant in HR^+^/HER2^−^ BC was 37.5% [350]. In the phase III SANDPIPER trial, taselisib showed a modest improvement (mPFS: 7.4 vs. 5.4 months) and demonstrated benefits in secondary efficacy endpoints among patients with *PIK3CA* mutations [351]. Despite its targeted approach, treatment with taselisib is associated with an elevated risk of severe side effects, including diarrhea, hyperglycemia, pneumonia, and colitis [352, 353].

##### 5.1.2.4 Capivasertib

Capivasertib (AZD5363) is an orally active Akt inhibitor that targets and suppresses the activity of all Akt isoforms [354]. It exhibited substantial anticancer effects in individuals with solid tumors carrying *Akt1* ^E17K^ mutations in a phase I trial [355]. Their combination with fulvestrant, capivasertib led to a notable enhancement in PFS compared with fulvestrant alone in individuals with HR^+^ advanced BC [356]. This benefit was observed in patients whose disease had progressed following treatment with an AI in the presence or absence of CDK4/6 inhibitor [357]. In the phase II FAKTION trial, the combination of capivasertib and fulvestrant significantly extended mPFS to 10.3 versus 4.8 months with placebo plus fulvestrant in women with ER^+^/HER2^−^ ABC resistant to AIs [358]. In contrast, open‐label phase I/II BEECH trial showed no PFS benefit (10.9 vs. 8.4 months) with capivasertib plus paclitaxel compared with placebo plus paclitaxel in ER^+^/HER2^−^ ABC or MBC [359]. However, in the phase III CAPItello‐291 trial, capivasertib plus fulvestrant again demonstrated a significant PFS improvement (7.2 vs. 3.6 months) over placebo plus fulvestrant in HR^+^/HER2^−^ ABC patients who had relapsed or progressed disease after AI therapy, with or without prior CDK4/6 inhibitor use [360]. Based on the positive outcomes of the CAPItello‐291 trial, the US FDA approved capivasertib in November 2023 for use alongside fulvestrant in adults with HR^+^/HER2^−^ LABC or MBC harboring *PIK3CA*, *Akt1*, or *PTEN* alterations, after at least one endocrine‐based therapy in the metastatic setting or recurrence within 12 months of completing adjuvant therapy [361]. Currently, the phase III open‐label, randomized CAPItello‐292 trial is ongoing to evaluate capivasertib in combination with a CDK4/6 inhibitor and fulvestrant versus CDK4/6 inhibitor plus fulvestrant in 895 patients with HR^+^/HER2^−^ LABC or MBC [362]. The most frequently reported side effects associated with the use of capivasertib include neutropenia, diarrhea, rash, and hyperglycemia [363, 364].

##### 5.1.2.5 Ipatasertib

Ipatasertib (GDC‐0068) is an orally active Akt inhibitor that competes with ATP for binding [365]. Currently, it is undergoing clinical trials for HR^+^ MBC, together with paclitaxel, CDK4/6 inhibitors, ET, and immunotherapy [366, 367]. Notably, a case study reported a notable clinical response in a heavily pretreated patient with HR^+^/HER2^−^ MBC carrying the *Akt1* ^E17K^ mutation when treated with fulvestrant and ipatasertib [368]. IPATunity130 was a randomized, double‐blind, phase III trial that evaluated ipatasertib plus paclitaxel versus placebo plus paclitaxel in 579 patients with PI3K pathway‐mutated HR^+^/HER2^−^ LABC or MBC who were unsuitable for endocrine‐based therapy [369]. This study showed no improvement in PFS or ORR, with both arms reporting a mPFS of 9.3 months [370]. There are two ongoing trials to further assess ipatasertib for the possible treatment of HR^+^/HER2^−^ BC. The FINER trial is a randomized, double‐blind, phase III study using ipatasertib plus fulvestrant versus placebo plus fulvestrant in ER^+^/HER2^−^ BC with 250 patients who progressed after first‐line CDK4/6 inhibitor and AI therapy [371]. The FAIM trial is a multicenter, randomized, open‐label phase II study evaluating the combination of ipatasertib, fulvestrant, and a CDK4/6 inhibitor in 324 patients with HR^+^/HER2^−^ MBC [372]. Commonly observed adverse events in patients treated with ipatasertib include diarrhea, fatigue, neutropenia, and peripheral neuropathy [373].

##### 5.1.2.6 Everolimus

Everolimus, a derivative of sirolimus, acts as an mTOR inhibitor by allosterically binding to the mTORC1 complex, thereby blocking downstream signaling in the PI3K/AKT/mTOR pathway [374]. In 2012, the US FDA and the European Medicines Agency (EMA) approved everolimus for the treatment of ER^+^ and HER2^−^ ABC following progression of AI therapy, marking it as the first inhibitor of the PI3K/Akt/mTOR axis approved for this indication [375]. This approval was based on the results of the phase III BOLERO‐2 trial, which demonstrated significantly prolonged mPFS with everolimus and exemestane combination compared with placebo plus exemestane in postmenopausal women with HR^+^/HER2^−^ABC who experienced recurrence or progression during or after AI therapy (mPFS: 7.8 vs. 3.2 months; mOS: 31.0 vs. 26.6 months) [376]. Consistent with BOLERO‐2, the international multicenter BOLERO‐5 trial reported a mPFS of 7.4 months, further validating the use of everolimus plus exemestane in postmenopausal Chinese patients with ER^+^/HER2^−^ ABC [375]. Similarly, in the BOLERO‐4 trial, the combination of everolimus and letrozole achieved a mPFS of 22 months in postmenopausal women with HR^+^/HER2^−^ ABC [377]. The reported adverse outcomes of everolimus include anemia, fatigue, hyperglycemia, rash, dyspnea, pneumonitis, stomatitis, and myelosuppression [378, 379].

##### 5.1.2.7 Gedatolisib

**FIGURE 7:**
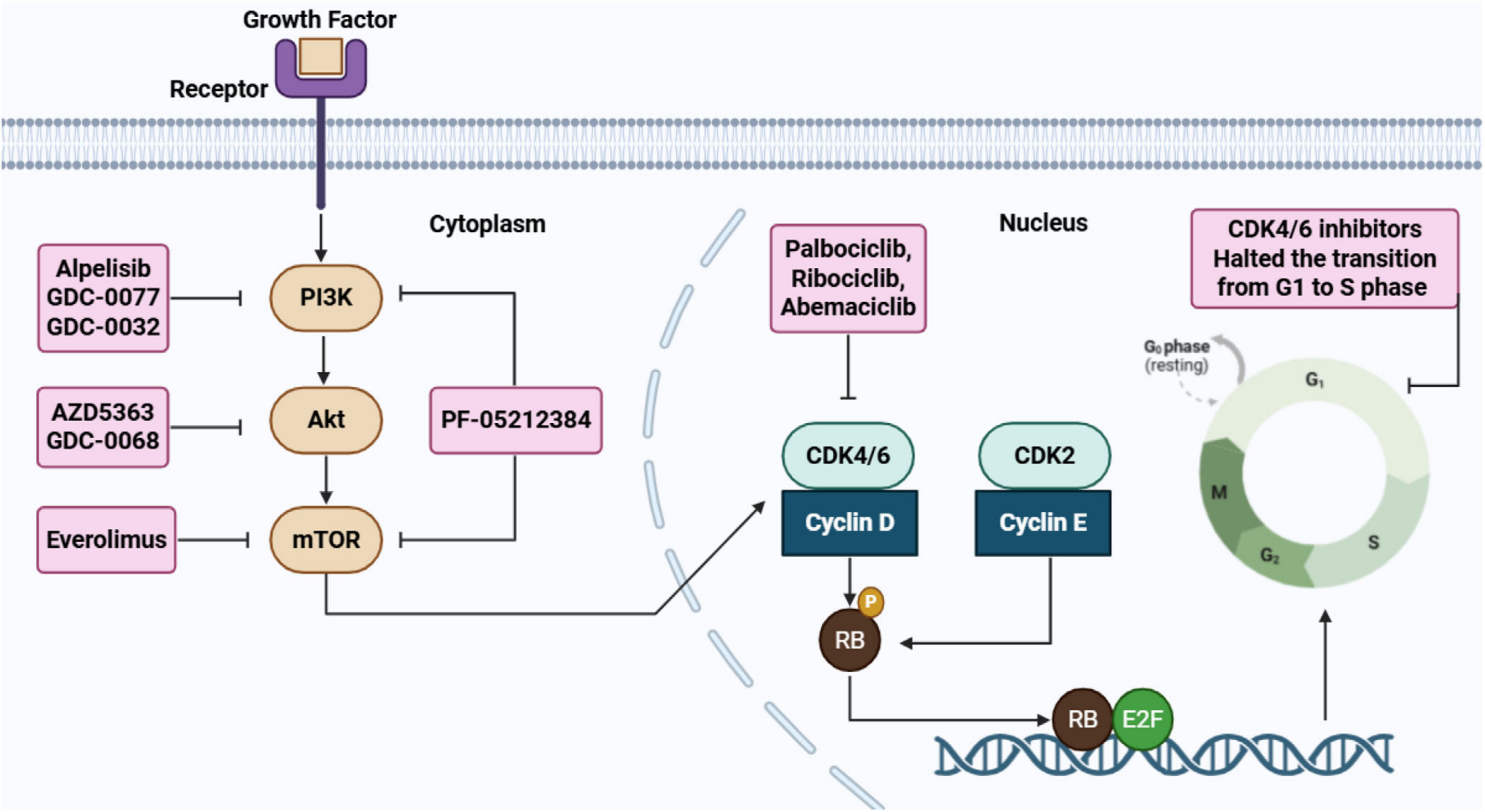
CDK4/6 inhibitors and PI3K/AkT/mTOR inhibitors in treatment of HR^+^/HER2^−^ BC. Inavolisib: GDC‐0077, taselisib: GDC‐0032, capivasertib: AZD5363, ipatasertib: GDC‐0068, gedatolisib: PF‐05212384. Akt, serine/threonine kinase; BC, breast cancer; mTOR, mammalian target of rapamycin; PI3K, phosphatidylinositide 3‐kinase. [Created using BioRender.com.]

Gedatolisib is a dual inhibitor of class I PI3Ks and mTOR [380]. A phase Ib trial involving HR^+^/HER2^−^ ABC patients treated with a combination of gedatolisib, palbociclib, and ET resulted in an ORR of 85.2% in treatment‐naïve patients, 61.5% in those previously treated but CDK4/6 inhibitor‐naïve, and 25–55.6% in patients with prior treatment with CDK4/6 inhibitors [381]. In addition, a phase Ib study also evaluated the potential of a combination of gedatolisib, palbociclib, and fulvestrant in HR^+^/HER2^−^ ABC, reporting a mPFS of 12.9 months in patients previously treated with CDK4/6 inhibitors [382]. Importantly, the clinical benefit was observed regardless of the PIK3CA mutation status. A phase III open‐label randomized trial (VIKTORIA‐1) is currently ongoing to assess the efficacy and safety of gedatolisib plus fulvestrant, with or without palbociclib, in patients with HR^+^/HER2^−^ LABC or MBC following the progression of CDK4/6 inhibitors and AIs [383]. Common adverse effects of gedatolisib include neutropenia, hyperglycemia, stomatitis, mucosal inflammation, rash, decreased appetite, diarrhea, fatigue, dysgeusia, and vomiting [381]. Table 4 summarizes various clinical trials evaluating PI3K/Akt/mTOR inhibitors for the treatment of HR^+^/HER2^−^ BC. Figure 7 illustrates the use of CDK4/6 and PI3K/AKT/mTOR inhibitors for HR^+^/HER2^−^ BC treatment.

**TABLE 4:** Clinical trials of PI3K/Akt/mTOR inhibitors for HR^+^/HER2^−^ BC treatment.

Target/drugs	Clinical trials (phase)/line of therapy	Patients	Treatment	Number of enrolments	Median PSF (months)	NCT ID	References
PI3K/alpelisib	SOLAR‐1 (III)/second or later	Postmenopausal women with HR^+^/HER2^−^ ABC or MBC, *PIK3CA*‐mutant	Alpelisib + fulvestrant vs. placebo + fulvestrant	572	11.0 vs. 5.7	NCT02437318	[336]
PI3K/inavolisib	INAVO120 (III)/first	*PIK3CA*‐mutant, HR^+^/HER2^−^ LABC or MBC	Inavolisib + palbociclib + fulvestrant vs. placebo + palbociclib + fulvestrant	321	15.0 vs. 7.3	NCT04191499	[343]
PI3K/inavolisib	INAVO121 (III)/second or third	*PIK3CA*‐mutant, HR^+^/HER2^−^ LABC or MBC	Inavolisib + fulvestrant vs. alpelisib + fulvestrant	400	Ongoing	NCT05646862	[345]
PI3K/taselisib (GDC‐0032)	SANDPIPER (III)/second or later	*PIK3CA*‐mutant, ER^+^/HER2^−^ ABC or MBC	Taselisib + fulvestrant vs. placebo + fulvestrant	629	7.4 vs. 5.4	NCT02340221	[351]
Akt/capivasertib	BEECH (I/II)/second or later	*PIK3CA*‐mutant, ER^+^/HER2^−^ ABC or MBC	Capivasertib + paclitaxel vs. placebo + paclitaxel	148	10.9 vs. 8.4	NCT01625286	[359]
Akt/capivasertib	CAPItello‐291 (III)/second or later	HR^+^/HER2^−^ LABC or MBC	Capivasertib + fulvestrant vs. placebo + fulvestrant	818	7.2 vs. 3.6	NCT04305496	[360]
Akt/capivasertib	CAPItello‐292 (III)/first	HR^+^/HER2^−^ LABC or MBC	Capivasertib + CDK4/6 inhibitors + fulvestrant vs. CDK4/6 inhibitors + fulvestrant	895	Ongoing	NCT04862663	[362]
Akt/capivasertib	FAKTION (II)/second or later	ER^+^/HER2^−^ ABC or MBC	Capivasertib + fulvestrant vs. placebo + fulvestrant	149	10.3 vs. 4.8, mOS: 29·3 vs. 23.4	NCT01992952	[358]
Akt/ipatasertib	IPATunity130 (III)/second or later	HR^+^/HER2^−^ LABC or MBC	Ipatasertib + paclitaxel vs. placebo + paclitaxel	579	9.3 vs. 9.3	NCT03337724	[370]
Akt/ipatasertib	FINER (III)/second or later	ER^+^/HER2^−^ ABC	Ipatasertib + fulvestrant vs. placebo + fulvestrant	250	Ongoing	NCT04650581	[371]
Akt/ipatasertib	FAIM (II)/first	HR^+^/HER2^−^ MBC	Ipatasertib + CDK4/6 inhibitor + fulvestrant vs. CDK4/6 inhibitor + fulvestrant	324	Ongoing	NCT04920708	[372]
mTOR/everolimus	BOLERO‐2 (III)/second or later	HR^+^/HER2^−^ LABC or MBC	Everolimus + exemestane vs. placebo + exemestane	724	7.8 vs. 3.2, mOS: 31.0 vs. 26.6	NCT00863655	[376]
mTOR/everolimus	BOLERO‐4 (II)/first	ER^+^/HER2^−^ LABC or MBC	Everolimus + letrozole	202	22	NCT01698918	[377]
mTOR/everolimus	BOLERO‐5 (II)/second or later	ER^+^/HER2^−^ LABC or MBC	Everolimus + exemestane vs. placebo + exemestane	159	7.4 vs. 2.0	NCT03312738	[375]
PI3K–mTOR/gedatolisib	VIKTORIA‐1 (III)/second or later	*PIK3CA*‐mutant, ER^+^/HER2^−^ ABC or MBC	Gedatolisib + fulvestrant +/− palbociclib vs. alpelisib + fulvestrant	701	Ongoing	NCT05501886	[383]

### 5.2 HER2‐Positive BC

#### 5.2.1 Monoclonal Antibodies

mAbs have significantly advanced the management of HER2^+^ BC by precisely targeting the HER2 receptor to inhibit tumor progression and improve patient outcomes [384]. mAbs such as trastuzumab, pertuzumab, and margetuximab specifically target and bind to the extracellular domain of HER2, thereby disrupting its signaling cascade and subsequently exerting a potent antitumor effect [385]. These antibodies also promote immune responses through antibody‐dependent cellular cytotoxicity (ADCC) and antibody‐dependent cell phagocytosis (ADCP) [386]. They achieve their potency by engaging immune cells, including NK cells, which target and destroy HER2^+^ cancer cells [387]. These antibodies also block the dimerization of HER2, preventing it from pairing with other receptors such as HER3 and HER1 and stopping the amplification of oncogenic signaling pathways [388, 389]. These antibodies effectively disrupted the activation of key signaling cascades, including PI3K/Akt and MAPK, independent of ligand activation [390]. Through these mechanisms, HER2‐targeted mAbs not only inhibit tumor growth, but also strengthen the immune system's capacity to target and destroy cancer cells [391].

##### 5.2.1.1 Trastuzumab

Trastuzumab is a humanized mAb designed to selectively attract the HER2 receptor, thereby inhibiting the growth of HER2‐overexpressing cells [392]. In 1998, the US FDA granted approval for its use in treating patients with HER2^+^ BC [393]. Trastuzumab selectively binds to subdomain IV of the HER2 extracellular domain, inhibiting HER2 activity [394]. Its effects involve both extracellular and intracellular activities [395]. Key intracellular mechanisms include blocking HER2‐driven signaling pathways, preventing proteolytic cleavage of HER2's extracellular domain, and decreasing the levels of proangiogenic factors [396]. In addition to inhibiting the HER2 signaling pathway, which results in cell cycle arrest, trastuzumab triggers ADCC and ADCP [397]. These processes are mediated by the interaction of trastuzumab's Fc portion with Fcγ receptors on immune effector cells, including NK cells and macrophages [398]. The effectiveness of trastuzumab can be affected by several factors such as the expression levels of HER2 in tumors, the existence of additional mutations or changes in the key signaling network, and the gradual onset of resistance mechanisms over time [399, 400]. Trastuzumab has significantly improved patient outcomes in HER2^+^ BC compared with conventional chemotherapy regimens [395]. The phase III BCIRG‐006 trial assessed trastuzumab as an adjuvant therapy for high‐risk early BC (EBC), comparing anthracycline‐based and nonanthracycline‐based regimens with standard treatment [401]. Adding trastuzumab to adjuvant therapy markedly improved both disease‐free and OS at the 5‐ and 10‐year follow‐ups, with no difference in efficacy between anthracycline‐containing and nonanthracycline regimens [402]. Furthermore, the NCCTG N9831 and NSABP B31 trials in EBC demonstrated a 4‐year DFS rate of 85.7% with trastuzumab plus chemotherapy compared with 73.7% for chemotherapy alone, and OS was found to be 93 versus 85.6%, respectively, [403]. A significant side effect of trastuzumab treatment is a greater probability of cardiotoxicity and congestive heart problems, which is linked to a reduction in left ventricular ejection fraction, highlighting the importance of careful cardiac monitoring during treatment [404, 405].

##### 5.2.1.2 Pertuzumab

Pertuzumab is a humanized mAb widely used in the treatment of HER2^+^ BC [406]. In 2017, the US FDA approved its use in combination with trastuzumab and chemotherapy as adjuvant therapy for patients with early stage HER2^+^ BC who were at a high risk of disease recurrence [393, 407]. Unlike trastuzumab, which targets subdomain IV of the HER2 extracellular domain, pertuzumab specifically binds to subdomain II, a critical site for receptor heterodimerization with other members of the EGFR family such as HER3 and HER1 [394, 408]. This binding effectively blocks HER2 heterodimerization, thereby effectively blocking the downstream activation of proliferative and survival pathways such as PI3K/Akt and MAPK, both of which are central to tumor development and progression [409]. Similar to trastuzumab, pertuzumab can also bind to Fc receptors on NK cells to induce ADCC and ADCP, thereby amplifying its antitumor immune response [398, 410]. The combined use of trastuzumab and pertuzumab is particularly effective because of their complementary HER2 receptor binding sites. This dual blockade not only enhances the inhibition of HER2‐mediated signaling but also improves clinical efficacy, making the trastuzumab–pertuzumab–taxane combination the standard first‐line therapy for HER2^+^ BC [411, 412]. Interestingly, a recent study suggested that this combination remains therapeutically effective even in the absence of taxanes, offering greater flexibility in treatment design [413]. Preliminary studies have evaluated the integration of pertuzumab and trastuzumab with radiation therapy, demonstrating that the combined regimen is generally well tolerated in HER2^+^ BC patients [414, 415]. Trastuzumab and pertuzumab bind cooperatively to the HER2 receptor, and their combined use has shown synergistic antitumor effects in preclinical studies, supporting their possible use in HER2^+^ BC [406]. The CLEOPATRA trial demonstrated a mOS of 57.1 months with dual HER2‐targeted therapy plus chemotherapy; almost 50% of the patients progressed well within 18 months and up to 80% within 5 years [416]. While dual HER2 blockade proved superior as a first‐line treatment for MBC, it did not show a significant benefit in second‐line treatment [417]. In the APHINITY trial, the dual binding of trastuzumab and pertuzumab effectively inhibited HER2 dimerization and improved outcomes in HER2^+^ EBC, with 6‐year DFS reaching 91% and OS 95% [418]. Similarly, in neoadjuvant settings, targeted therapy has shown significant clinical benefits [419]. The phase II NeoSphere trial evaluated combinations of pertuzumab, trastuzumab, and docetaxel, revealing the highest pathological complete response (pCR) rate of 46% in the group receiving all three agents, whereas other regimens achieved pCR rates between 17 and 29% [420]. However, this study did not establish a PFS advantage for the pertuzumab–trastuzumab–docetaxel combination, which could be due to the limited sample size [421]. Furthermore, the phase III TRAIN‐2 trial demonstrated that dual HER2 blockade with chemotherapy increased pCR rates up to 68%, with a 3‐year OS rate of 97.7% [422]. Reported adverse effects are typically mild and include asymptomatic decreases in the left ventricular ejection fraction, mild radiation‐induced pneumonitis, esophagitis, acute dermatologic reactions, and radiodermatitis [414, 423]. Importantly, no major cardiac complications were observed [424]. The most common side effects associated with pertuzumab include diarrhea, fatigue, skin rash, hypertension, and neutropenia, all of which are generally manageable in the clinical settings [425, 426].

##### 5.2.1.3 Margetuximab

Margetuximab, previously known as MGHA22, is a next‐generation mAb specifically engineered to target subdomain IV of the HER2 receptor, the same epitope as trastuzumab [427]. In 2020, the US FDA approved margetuximab for use in combination with chemotherapy to treat adults with HER2^+^ MBC (HER2^+^ MBC) who had previously received at least two HER2‐targeted treatment regimens [428]. The key innovation of margetuximab lies in its engineered Fc domain, which is designed to enhance binding to the activating Fcγ receptor CD16A while reducing affinity for the inhibitory Fcγ receptor CD32B [429]. This modification leads to a stronger and more sustained ADCC response, particularly through improved engagement of CD16A, a receptor variant that is less effectively bound by trastuzumab [430]. Approximately 85% of patients carry the lower‐affinity CD16A‐158F variant, making margetuximab a more effective therapeutic option in this genetic context [431]. The greater binding of margetuximab to CD16A‐158F provides therapeutic significance across other anti‐HER2 antibodies in BC treatment [432]. Moreover, the combination of margetuximab and chemotherapy has demonstrated a favorable safety profile and a notable increase in PFS when compared with chemotherapy plus trastuzumab [433]. This benefit has been observed in patients with HER2^+^ MBC who have shown disease progression despite undergoing at least two‐phase anti‐HER2 therapies [434]. The SOPHIA trial evaluated the efficacy of margetuximab versus trastuzumab in patients with advanced BC who had progressed after at least two prior HER2‐targeted treatments, including pertuzumab [435]. In the intention‐to‐treat population, margetuximab provided a modest improvement in PFS, extending it by about one month compared with trastuzumab [431]. The most commonly reported adverse effects of margetuximab include fatigue, fever, vomiting, nausea, diarrhea, neutropenia, constipation, and abdominal pain [427, 436]. Other common adverse reactions include headaches, hair loss, palmar–plantar erythrodysesthesia, peripheral neuropathy, cough, reduced appetite, limb pain, and infusion‐related complications [428, 437].

#### 5.2.2 Tyrosine Kinase Inhibitors

TKIs are small molecules designed to target the intracellular kinase domain of HER2, effectively blocking its activity and hindering tumor growth, proliferation, and survival [438]. Drugs, such as lapatinib, neratinib, tucatinib, and pyrotinib, function by binding to the ATP‐binding site of HER2, inhibiting the phosphorylation of kinases, and disrupting downstream signaling pathways [439]. This action can promote the apoptosis of cancerous cells, prevent tumor cell proliferation, disrupt the cell cycle, and slow tumor progression [440, 441].

##### 5.2.2.1 Lapatinib

Lapatinib is a powerful, reversible TKI that targets both HER1/EGFR and HER2 [442]. It received US FDA approval in 2007 for treating patients with HER2^+^ MBC [443]. Lapatinib targets the cytoplasmic ATP‐binding site of TK on HER1, HER2/ErbB2, and EGFR [444]. By binding to this site, it suppresses receptor phosphorylation and activation, thereby inhibiting downstream signaling pathways, such as PI3K/Akt and ERK‐1/2 [445]. This drug is commonly prescribed for the increased risk of HER2^+^ MBC in patients who have undergone earlier therapies, including anthracyclines, taxanes, and trastuzumab, or in those who have become resistant to trastuzumab [446, 447]. The EU has approved the combination of lapatinib and trastuzumab for patients with HR^+^/HER2^+^ MBC who have already been treated with trastuzumab [448]. The phase III MA.31 (NCT00667251) trial compared lapatinib and trastuzumab, each combined with a taxane, as first‐line treatments for HER2^+^ MBC. Results showed that lapatinib was associated with a 20% reduction in PFS and higher toxicity, confirming the superior efficacy and safety of trastuzumab [449]. The ALTERNATIVE trial assessed lapatinib versus trastuzumab in both first‐ and later‐line settings for HR^+^/HER2^+^ MBC. Patients were assigned to one of the following three groups: lapatinib plus an AI, trastuzumab plus AI, or a combination of lapatinib and trastuzumab with AI. The dual blockade arm was found to have nearly doubled mPFS from 5.6 to 11 months compared with the trastuzumab arm. Although mOS improved, the difference was not statistically significant [450]. The most common adverse events of lapatinib include diarrhea, vomiting, nausea, fatigue, abdominal cramping, skin rashes, hand–foot syndrome, dyspepsia, and gastroesophageal reflux disease [423, 451].

##### 5.2.2.2 Neratinib

Neratinib is an irreversible chloroanilino‐quinazoline derivative and potent inhibitor of HER2 [452]. In 2017, the US FDA approved it for adjuvant therapy in patients with stages I–III HER2^+^ BC who had already completed 1 year of adjuvant trastuzumab treatment [453]. In a preclinical study, neratinib demonstrated potent and selective inhibition of HER2^+^ cell line proliferation [454]. It effectively reduced autophosphorylation of the HER2 receptor, subsequently blocking downstream signaling through the PI3K/Akt and MAPK pathways [455]. The ExteNET trial in HER2^+^ EBC patients who had completed trastuzumab‐based therapy, neratinib, improved the 5‐year invasive DFS (iDFS) from 87.7 to 90.2% [456]. In the NALA trial, neratinib and lapatinib were evaluated in combination with capecitabine as third‐line treatments for metastatic HER2^+^ BC in patients previously treated with at least two HER2‐targeted regimens. Neratinib arm demonstrated a slight but statistically significant advantage in mPFS (5.6 vs. 5.5 months) and mOS (21.0 vs. 18.7 months) compared with lapatinib arm [457]. The most frequently recorded negative effects of this drug include nausea, vomiting, diarrhea, and fatigue, while fewer cases of central nervous system problems have been observed [458, 459].

##### 5.2.2.3 Tucatinib

Tucatinib is a reversible TKI that specifically targets HER2 and exhibits minimal inhibition of EGFR [460]. This selectivity contributes to a more favorable tolerability profile than that of other TKIs [461]. In April 2020, the US FDA approved tucatinib alongside trastuzumab and capecitabine for treating HER2^+^ MBC patients [462]. This approval included patients with brain metastases who had previously received at least one anti‐HER2 therapy in the metastatic state, but did not achieve successful outcomes [463]. However, the specific mechanism of action of tucatinib is not completely understood [464]. However, molecular studies suggest that it binds strongly and stably to the ATP pocket of HER2 TKs [465]. This binding prevents the phosphorylation of HER2, thereby blocking downstream signaling through the PI3K and MAPK pathways [441]. The phase II HER2CLIMB trial evaluated tucatinib as a third‐line therapy for advanced HER2^+^ BC after progression on trastuzumab, pertuzumab, and trastuzumab emtansine treatment. In this trial, patients received tucatinib or placebo with capecitabine and trastuzumab. The tucatinib regimen improved mPFS by 2.2 months and mOS by 4.5 months compared with placebo [466]. The most common adverse effects of tucatinib include nausea, vomiting, diarrhea, increased aminotransferase levels, and palmar–plantar erythrodysesthesia syndrome [467, 468].

##### 5.2.2.4 Pyrotinib

**FIGURE 8:**
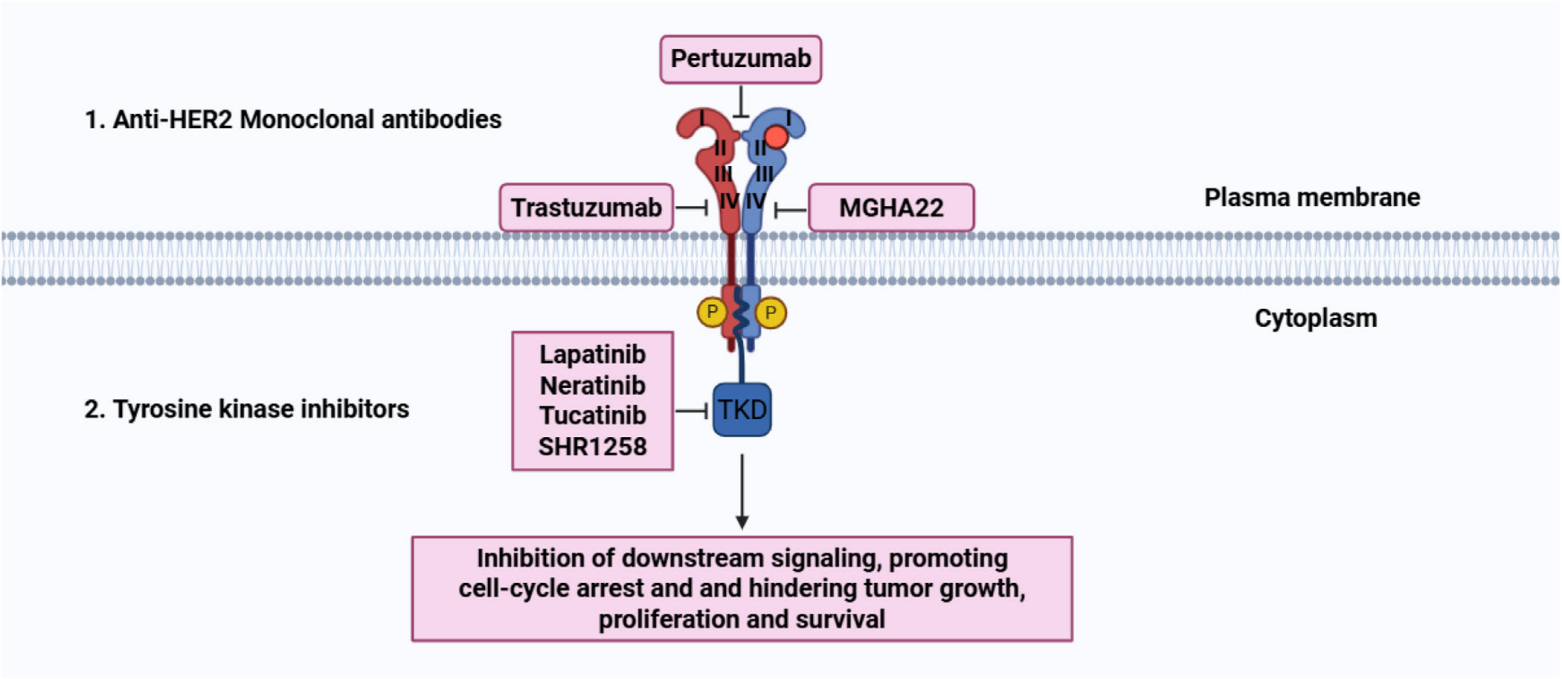
Monoclonal antibodies and TKIs in treatment of HER2^+^ BC. Margetuximab: MGHA22, pyrotinib: SHR1258. BC, breast cancer; TKD, tyrosine kinase domain; TKI, tyrosine kinase inhibitors. [Created using BioRender.com.]

Pyrotinib is an irreversible TKI that targets multiple HER family receptors [469]. In 2018, it was conditionally approved by the Chinese State Drug Administration for use along with capecitabine to treat patients with HER2^+^ MBC [470]. This approval was obtained for individuals who had previously undergone chemotherapy with anthracyclines or taxanes [471]. Recent investigations have shown the effectiveness of pyrotinib in neoadjuvant therapy, where it targets pan‐ErbB receptors by covalently binding to ATP‐binding sites within the intracellular kinase domains of HER [472]. This action suppresses HER dimer autophosphorylation, thereby blocking key signaling pathways, including PI3K/Akt and Ras/Raf/MEK/MAPK, which are important in tumor progression and development [469, 473]. The phase III PHOEBE trial in HER2^+^ MBC compared pyrotinib plus capecitabine with lapatinib plus capecitabine, showed mPFS of 12.5 versus 6.8 months, respectively [474]. Additionally, preliminary results from the PHILA phase III study suggest that combining pyrotinib, trastuzumab, and docetaxel may extend mPFS to 24.3 months in patients resistant to trastuzumab, although 23–33% of patients discontinued treatment due to disease progression [475]. The most frequently reported adverse events of pyrotinib include anemia, nausea, vomiting, diarrhea, reduced neutrophil count, and hand–foot syndrome [476, 477]. Table 5 presents the clinical trials evaluating mAbs and TKIs for HER2^+^ BC treatment. Figure 8 illustrates the use of mAbs and TKIs for the treatment of HER2^+^ BC.

**TABLE 5:** Clinical trials investigating the monoclonal antibodies and TKIs for HER2^+^ BC treatment.

Trial name/year	Patients	Regimens	Treatment phase/follow‐up period	DFS/PFS	OS	NCT ID	References
BCIRG‐006/2011	HER2^+^ EBC	AC‐TH vs. TCbH vs. AC‐T	Adjuvant therapy/5 years	DFS: 84 vs. 81 vs. 75%	92 vs. 91 vs. 87%	NCT00021255	[401]
NCCTG N9831 and NSABP B31/2011	HER2^+^ EBC	AC‐TH vs. AC‐T	Adjuvant therapy/4 years	DFS: 85.7 vs. 73.7%	93 vs. 85.6%	NCT00004067 and NCT00005970	[403]
CLEOPATRA/2012	HER2^+^ MBC	THP vs. TH	Advance‐stage treatment	mPFS: 18.5 vs. 12.4	mOS: 57.1 vs. 40.8	NCT00567190	[416]
APHINTY/2017	HER2^+^ EBC	HP + CT vs. H + CT	Adjuvant therapy/6 years	DFS: 91 vs. 88%	95 vs. 94%	NCT01358877	[418]
Neosphere/2012	HER2^+^LABC IBC EBC	HT* 4‐FEC* 3 vs. HPT* 4‐FEC* 3 vs. HP* 4‐FEC* 3 vs. PT* 4‐FEC* 3	Adjuvant therapy/5 years	DFS: 81 vs. 84 vs. 80 vs. 75%	—	NCT00545688	[420]
TRAIN−2/2018	HER2^+^ EBC	TCbHP* 9 vs. FECHP* 3‐TCbHP* 6	Adjuvant therapy/3 years	DFS: 92.7 vs. 93.6%	97.7 vs. 98.2%	NCT01996267	[422]
SOPHIA/2020	HER2^+^ MBC	MCT vs. HCT	Advance‐stage treatment/3 years	mPFS: 5.8 vs. 4.9	mOS: 21.6 vs. 19.8	NCT02492711	[435]
MA. 31/2015	HER2^+^ MBC	LTax vs. HTax	Advance‐stage treatment/3 years	mPFS: 9.0 vs. 11.3	mOS: 20.8 vs. 21.9	NCT00667251	[449]
ALTERNATIVE/2016	HR^+^/HER2^+^ MBC	LHAI vs. HAI	Advance‐stage treatment	mPFS: 11.0 vs. 5.6	mOS: 46.0 vs. 40.0	NCT01160211	[450]
ExteNET/2020	HER2^+^ EBC	N vs. Pla	Adjuvant therapy/5 years	iDFS: 87.7 to 90.2%	94.09 vs. 93.26%	NCT00878709	[456]
NALA/2020	HER2^+^ MBC	N + Cap vs. L + Cap	Advance‐stage treatment	mPFS: 5.6 vs. 5.5	mOS: 21.0 vs. 18.7	NCT01808573	[457]
PHOEBE/2021	HER2^+^ MBC	Pyr + Cap vs. L + Cap	Advance‐stage treatment	mPFS: 12.5 vs. 6.8	—	NCT03080805	[474]
PHILA/2023	HER2^+^ MBC	Pyr + H + T vs. Pla + H + T	Advance‐stage treatment	mPFS: 24.3 vs. 10.4	—	NCT03863223	[475]
HER2CLIMB/2020	HER2^+^ MBC	Tuc + Cap + H vs. Pla + Cap + H	Advance‐stage treatment	mPFS: 7.8 vs. 5.6	mOS: 21.09 vs. 17.4	NCT02614794	[466]

Abbreviations: A, doxorubicin/epirubicin; AI, aromatase inhibitor; C, cyclophosphamide; Cap, capecitabine; Cb, carboplatin; CT, chemotherapy; EBC, early breast cancer; F, fluorouracil (5‐FU); H, trastuzumab; IBC, inflammatory breast cancer; L, lapatinib; LABC, locally advanced breast cancer; M, margetuximab; MBC, metastatic breast cancer; N, neratinib; P, pertuzumab; Pla, placebo; Pyr, pyrotinib; T, docetaxel/paclitaxel; Tax, taxane; Tuc, tucatinib.

### 5.3 Triple‐Negative BC

#### 5.3.1 PARP Inhibitors

PARP enzymes are crucial for various cellular processes, particularly the repair of single‐strand DNA breaks via the base excision repair pathway [478]. PARP1 is the most studied member of this family, accounting for 85–90% of the total PARP activity [479]. When PARP is inhibited, PARP–DNA complexes become trapped at the replication fork, ultimately leading to the conversion of single‐strand breaks into double‐strand breaks, which subsequently inducing apoptosis [480]. Approximately 10–15% of individuals with TNBC carry germline mutations in *BRCA1/2* tumor suppressor genes, which are crucial for the repair of double‐strand DNA breaks [481]. Additionally, cancer cells carrying *BRCA* mutations often adapt to genomic instability by upregulating PARP enzyme expression [482]. Therefore, PARP inhibitors have shown significant effectiveness in treating *BRCA*‐mutated subtypes of *BRCA*‐mutated TNBC [483]. Furthermore, these inhibitors have also been associated with improved PFS in *BRCA* mutated BC patients [484]. PARP inhibitors, such as olaparib, talazoparib, and veliparib, are effectively used to treat metastatic TNBC (mTNBC) with *BRCA*‐positive mutations or deletions following chemotherapy [485].

##### 5.3.1.1 Olaparib (AZD2281)

Olaparib is a member of the N‐acyl piperazine class and the first PARP inhibitor authorized by US FDA [486]. A phase I trial evaluated the potential of the oral PARP inhibitor olaparib combined with weekly paclitaxel for first‐ or second‐line treatment of mTNBC. However, the combination was associated with significant drug interaction, leading to unexpectedly high rates of neutropenia despite the use of secondary prophylaxis [487]. In the phase II PETREMAC trial, patients with primary TNBC were treated with olaparib for up to 10 weeks prior to chemotherapy, which resulted in a high clinical response and minimal side effects [488]. In the nonmetastatic setting, olaparib has been approved as an adjuvant therapy for HER2^−^ germline *BRCA1/2*‐mutated primary BC following definitive local treatment and neoadjuvant or adjuvant chemotherapy. This approval was based on the OlympiA trial, which demonstrated improved 3‐year iDFS with olaparib compared with placebo (85.9 vs. 77.1%). Notably, most participants (82.2%) had TNBC, and subgroup analysis confirmed a greater benefit in TNBC than in HR^+^ BC [489]. Although olaparib is currently used mainly as a monotherapy, ongoing clinical trials are assessing its effectiveness in combination with other established treatments. In a phase I/Ib study involving women with TNBC, the combination of olaparib and carboplatin was found to be tolerable and showed modest benefits in patients with sporadic TNBC [490]. Additionally, a phase Ib clinical trial evaluating alpelisib combined with olaparib in patients with advanced TNBC reported a disease control rate (DCR) of 77% [491]. The most frequently observed side effects in patients treated with olaparib include nausea, vomiting, diarrhea, anemia, neutropenia, fatigue, leukopenia, respiratory tract infections, joint and muscle pain, headache, indigestion, dysgeusia, reduced appetite, constipation, and mouth sores [492, 493].

##### 5.3.1.2 Talazoparib (BMN‐673)

Talazoparib is an oral PARP inhibitor that competes with the catalytic sites of PARP1 and PARP2 [494]. In 2018, the US FDA approved it for treating patients with HER2^−^, advanced, or MBC associated with deleterious or suspected deleterious germline *BRCA* mutations [495]. This drug demonstrates highly effective anticancer activity, showing 20–200 times better efficacy in vitro than other PARP inhibitors [496]. Its effectiveness is particularly notable in tumor cells deficient in *BRCA1*, *BRCA2*, or *PTEN* [497]. It exhibits high potency in cell cultures owing to its ability to effectively trap PARP–DNA complexes, a process linked to cancer cell death [498]. The EMBRACA trial compared talazoparib with standard chemotherapy and demonstrated a significantly longer PFS in the PARP inhibitor group (8.6 vs. 5.6 months). Unlike the OlympiAD trial, EMBRACA did not reveal a distinct improvement in the hazard ratio for TNBC in the subgroup analysis [499]. The phase II NEOTALA trial investigated neoadjuvant talazoparib in patients with early‐stage TNBC with germline *BRCA1/2* mutations. The pCR rate was 45.8% in the evaluable group and 49.2% in the intent‐to‐treat (ITT) population, comparable with the pCR rates typically achieved with anthracycline‐ and taxane‐based regimens [500]. In the KCSG BR21‐10 phase II trial, talazoparib was assessed as a maintenance therapy in advanced TNBC patients regardless of *BRCA* mutation status following platinum‐based chemotherapy, with mPFS in the placebo group expected to be 3 months from randomization [501]. Talazoparib prescription guidelines highlight important warnings and precautions regarding the potential risks of acute myeloid leukemia, myelodysplastic syndrome, myelosuppression, and embryo‐fetal toxicity [502]. The most common side effects observed in patients using talazoparib include diarrhea, fatigue, nausea, vomiting, anemia, neutropenia, headache, hair loss, thrombocytopenia, and decreased appetite [503, 504].

##### 5.3.1.3 Veliparib (ABT‐888)

Veliparib is a novel PARP inhibitor with acceptable toxicity but has not yet been approved by the US FDA. It has been widely investigated in combination with other chemotherapeutic agents [505]. In a phase I clinical trial, its combination with cisplatin and vinorelbine, a microtubule‐destabilizing agent, demonstrated an ORR of 73% in TNBC patients harboring *BRCA1/2* mutations [506]. Additionally, the phase II I‐SPY 2 trial (NCT01042379), a multicenter study, showed increased pCR rates in patients with TNBC treated with veliparib and carboplatin. Specifically, the pCR rate was 51% in the veliparib–carboplatin group compared with 26% in the control group [507]. However, studies investigating the addition of veliparib to carboplatin and standard chemotherapy in stages IIb–IIIc in TNBC (NCT01818063), as well as its combination with cisplatin in *BRCA* mutated BC and/or mTNBC (NCT02595905), have been completed, and the results remain unpublished [508]. In 2018, findings from the phase III trial of BrighTNess (NCT02032277) were released, evaluating veliparib with paclitaxel and carboplatin as part of the standard neoadjuvant chemotherapy for TNBC. Although several toxicities and serious adverse effects were frequently associated with carboplatin, veliparib did not significantly contribute to existing toxicity. The most frequently reported adverse events throughout this treatment included neutropenia, anemia, thrombocytopenia, and febrile neutropenia [509]. A phase I trial (ETCTN 8620) investigated veliparib in combination with weekly carboplatin and paclitaxel in patients with advanced solid tumors, including an expansion cohort for TNBC. Among 23 patients with evaluable disease, the ORR was 65%, whereas in 19 evaluable TNBC patients, the ORR was 63%. The most frequently observed adverse events were anemia, neutropenia, and thrombocytopenia [510].

##### 5.3.1.4 Niraparib

Niraparib is a PARP1 and PARP2 inhibitor that has been approved for maintenance therapy in patients with recurrent cancers, particularly those exhibiting HR deficiency (HRD) [511]. An open‐label phase II clinical trial combining niraparib with pembrolizumab for advanced or mTNBC reported a DCR of 80% in patients with *BRCA* mutations compared with 44% in those with *BRCA* wild‐type [512]. In addition, a phase I trial (NCT03945721) assessing adjuvant niraparib alongside postoperative radiation therapy in localized TNBC showed hematologic toxicities in 24% of patients, while 19% experienced dermatitis. Reported adverse events associated with niraparib include dermatitis, thrombocytopenia, anemia, lymphopenia, and elevated alkaline phosphatase levels [513]. Moreover, a phase I study assessed the safety and tolerability of niraparib combined with everolimus in patients with advanced TNBC. Among the 11 evaluable patients, the ORR was 18% and the clinical benefit rate (CBR) was 45%. The mPFS was 6 months and the mOS was approximately 18 months. Frequently reported adverse effects included anemia, nausea, fatigue, thrombocytopenia, anorexia, neutropenia, constipation, vomiting, and elevated alkaline phosphatase [379].

#### 5.3.2 EGFR Inhibitors

EGFR is a transmembrane TK receptor, crucial for controlling cell growth, proliferation, and differentiation in normal tissues [514]. It also contributes to cell adhesion, movement, and activation of several downstream signaling cascades [515, 516]. It is overexpressed in approximately 70–78% of basal‐like TNBC cases and is associated with poor prognosis and chemotherapy resistance [517]. As a member of the ErbB family of membrane TK receptors, EGFR has been extensively studied in TNBC. The phase II BALI‐1 trial (NCT00463788), an open‐label randomized study, compared cisplatin plus the anti‐EGFR mAb cetuximab versus cisplatin alone in mTNBC. This combination achieved an ORR value of 20% compared with 10% for cisplatin alone, mPFS of 3.7 versus 1.5 months and mOS of 12.9 versus 9.4 months, respectively [518]. In a multicenter neoadjuvant phase II pilot study (NCT00600249), cetuximab combined with docetaxel in patients with operable TNBC showed an ORR of 57%, indicating modest efficacy [519]. In another phase II trial (NCT00633464), evaluating ixabepilone alone and in combination with cetuximab as first‐line therapy for advanced/mTNBC, the ORR was 30% with ixabepilone alone and 35.9% with the combination, and the mPFS was 4.1 months in both groups [520]. One study showed enhanced apoptosis and mitotic catastrophe in TNBC cells by a nanocomposite formulation of nanodiamond‐paclitaxel‐cetuximab through EGFR targeting, thereby suggesting a promising therapeutic approach [521]. Additionally, a multicenter phase II trial (NCT02833766) achieved an mPFS of 3.5 months with anti‐EGFR immunoliposomes loaded with doxorubicin in patients with advanced TNBC [522]. Furthermore, a preclinical study exhibited strong antitumor activity of afatinib across 14 TNBC cell lines, with IC_50_ values ranging from 0.008 to 5.0 µM, which is particularly effective in the basal‐like subtype TNBC. The combination of afatinib and dasatinib induced G1 cell cycle arrest and significantly inhibited pERK (T202/T204) and pAkt (S473) signaling, resulting in significant antiproliferative effects [523]. Moreover, in a phase II trial (NCT02593175), panitumumab combined with carboplatin and paclitaxel was evaluated as a second phase of neoadjuvant therapy in TNBC patients who did not respond to initial treatment with doxorubicin and cyclophosphamide. The primary endpoint of this study was a pCR/residual cancer burden class I (RCB‐I) rate of 30.2% compared with 5% in historical controls [524]. Frequently observed adverse events associated with EGFR inhibitor use include acne, rash, anemia, neutropenia, alopecia, fatigue, vomiting, constipation, and decreased appetite [518, 520].

#### 5.3.3 Antiangiogenesis and VEGF Inhibitors

Angiogenesis is the process of new blood vessel formation, which is crucial for the growth and spread of BC by delivering nutrients and oxygen to tumor cells. VEGF is a key signaling protein that promotes angiogenesis [525]. In TNBC, VEGF is significantly overexpressed compared with other BC subtypes [505]. Elevated VEGF levels are associated with reduced RFS and OS [526]. Angiogenesis inhibitors are a group of drugs designed to disrupt this process by depriving vital nutrients and oxygen to tumor cells, thereby slowing their growth [527]. These inhibitors mainly target VEGF and its receptors, which are crucial for blood vessel development in cancer [528].

Bevacizumab, a mAb targeting VEGF, has been investigated in clinical trials for its ability to suppress angiogenesis in metastatic and aggressive BC [529]. In a study evaluating first‐line bevacizumab‐containing therapy for TNBC, the ORR was 49%, with a median time to progression of 7.2 months and a mOS of 18.3 months [530]. Additionally, the phase III RIBBON‐2 trial (NCT00281697), which assessed second‐line bevacizumab in mTNBC, reported a mPFS of 6 months with bevacizumab plus chemotherapy, compared with 2.7 months with chemotherapy alone, mOS was 17.9 versus 12.6 months, and ORR was 41 versus 18%, respectively [531]. In the phase III GeparQuinto trial (NCT00567554), neoadjuvant bevacizumab combined with anthracycline–taxane‐based chemotherapy in primary TNBC increased the pCR rate from 27.9 to 39.3% [532]. In the multicenter phase II KCSG BR‐0905 trial, bevacizumab with docetaxel–carboplatin (neoadjuvant therapy) achieved a pCR rate of 42% in TNBC [533]. Similarly, the CALGB 40603 trial (NCT00861705) evaluated carboplatin or bevacizumab combined with neoadjuvant chemotherapy for stages II–III TNBC. Carboplatin improved both pCR breast and pCR breast/axilla rates, while bevacizumab increased only the pCR breast rate, and the combination produced the highest pCR rate, with no significant interaction between the agents [534]. However, in the phase III BEATRICE trial (NCT00528567), adding bevacizumab to adjuvant standard chemotherapy in early‐stage TNBC did not significantly improve OS. The OS rate was 88% in both groups, and the iDFS was 77% with chemotherapy alone versus 80% with bevacizumab [535]. In a phase II study (NCT01201265) evaluating first‐line treatment for mTNBC, bevacizumab combined with gemcitabine and carboplatin showed improved survival and an acceptable safety profile [536]. The phase II ATRACTIB trial (NCT04408118) assessed a combination of bevacizumab, atezolizumab, and paclitaxel in advanced TNBC, demonstrating promising clinical activity, notably higher mPFS, and a manageable safety profile [537]. Furthermore, a multicenter phase II study (NCT01176669) of the VEGFR inhibitor apatinib in mTNBC, the ORR and CBR were 10.7 and 25.0%, respectively, with mPFS of 3.3 months and mOS of 10.6 months [538]. In another open‐label phase II trial (NCT03394287), camrelizumab combined with apatinib in advanced TNBC achieved an ORR of 43.3% in the continuous dosing cohort, whereas no ORR was observed in the intermittent dosing cohort. The DCR was 63.3% with continuous dosing versus 40% with intermittent dosing, mPFS was 3.7 versus 1.9 months, respectively [539]. ENMD‐2076, an aurora‐A kinase inhibitor with antiangiogenic activity, has demonstrated the ability inhibits cell proliferation and induces apoptosis in TNBC preclinical models. In a single‐arm, two‐stage phase II trial (NCT01639248), ENMD‐2076 was administered to previously treated patients with advanced or mTNBC until disease progression or unacceptable toxicity. Among the 41 patients, partial responses were observed in two cases, and the 6‐month CBR was 16.7%, indicating a promising therapeutic potential [540]. Frequently reported negative effects of angiogenesis inhibitors include nausea, fatigue, diarrhea, skin rashes, bleeding, hypertension, thromboembolism, asymptomatic proteinuria, delayed wound healing, reversible leukoencephalopathy syndrome, gastrointestinal perforation, and hypersensitivity reactions related to infusion [541, 542]. Table 6 presents the clinical trials assessing the efficacy of PARP, EGFR, and VEGF inhibitors in TNBC treatment.

**TABLE 6:** Clinical trials of PARP, EGFR, and VEGF inhibitors in TNBC treatment.

Target/drug(s)	Trail stages (phase)	Patients	Treatment	Results	NCT ID	References
PARP/olaparib	PETREMAC (II)	Naïve TNBC, HR deficiency	Olaparib before chemotherapy	High clinical response rate associated with minor side‐effects	NCT02624973	[488]
PARP/olaparib	OlympiA (III)	BRCA 1/2 mutated BC	Olaparib vs. placebo	iDFS: 85.9 vs. 77.1%	NCT02032823	[489]
PARP/talazoparib	EMBRACA (III)	Metastatic TNBC	Talazoparib vs. physicians choice	mPFS: 8.6 months vs. 5.6 months	NCT01945775	[499]
PARP/talazoparib	NEOTALA (II)	*gBRCA1/2* mutated early‐stage TNBC	Talazoparib for neoadjuvant treatment	pCR: 45.8% in evaluable population and 49.2% in ITT population	NCT03499353	[500]
PARP/talazoparib	KCSG BR21‐10 (II)	*gBRCA1/2* mutated advanced TNBC	Talazoparib vs. placebo	mPFS: 3 months in the placebo maintenance arm	NCT04755868	[501]
PARP/veliparib	I‐SPY 2 (II)	TNBC	Veliparib with carboplatin	pCR: 51% vs. 26	NCT01042379	[507]
PARP/veliparib	BrighTNess (III)	TNBC	Veliparib combined with paclitaxel plus carboplatin	Veliparib did not substantially increased toxicity	NCT02032277	[509]
PARP/niraparib	TOPACIO (II)	Advanced or metastatic TNBC	Niraparib with pembrolizumab	DCR: 80% in *BRCA*‐mutated patients	NCT02657889	[512]
PARP/niraparib	UNITY (I)	Localized TNBC	Niraparib with radiation therapy	24% hematologic toxicities and 19% dermatitis	NCT03945721	[513]
PARP/niraparib	Phase I	Advanced TNBC	Niraparib with everolimus	mPFS: 6 months, mOS: 18 months	NCT03154281	[379]
EGFR/cetuximab	BALI‐1 (II)	Metastatic TNBC	Cetuximab with cisplatin vs. cisplatin	ORR: 20 vs. 10%, mPFS: 3.7 vs. 1.5, OS: 12.9 vs. 9.4	NCT00463788	[518]
EGFR/cetuximab	Phase II	Operable TNBC	Cetuximab combined with docetaxel	ORR of 57%, modest efficacy	NCT00600249	[519]
EGFR/cetuximab	Phase II	Advanced/metastatic TNBC	Ixabepilone alone or in combination with cetuximab	ORR: 30 vs. 35.9%, mPFS: 4.1 months in both groups	NCT00633464	[520]
EGFR/doxorubicin	Phase II	Advanced TNBC	Immunoliposomes loaded with doxorubicin	mPFS: 3.5 months	NCT02833766	[522]
EGFR/panitumumab	Phase II	TNBC	Panitumumab with carboplatin, and paclitaxel	pCR/RCB‐I rate: 5% compared with 20%	NCT02593175	[524]
VEGF/bevacizumab	RIBBON‐2 (III)	Metastatic TNBC	Bevacizumab–chemotherapy vs. chemotherapy alone	mPFS: 6 vs. 2.7 months, mOS: 17.9 vs. 12.6 months, and ORR: 41 vs. 18%, respectively	NCT00281697	[531]
VEGF/bevacizumab	GeparQuinto (III)	Primary TNBC	Bevacizumab to neoadjuvant anthracycline–taxane‐containing chemotherapy	Increased pCR rate from 27.9 to 39.3%	NCT00567554	[532]
VEGF/bevacizumab	Phase II	Metastatic TNBC	Bevacizumab in combination with gemcitabine and carboplatin	Improved survival outcomes and manageable safety profile	NCT01201265	[536]
VEGF/bevacizumab	ATRACTIB (II)	Advanced TNBC	Bevacizumab + atezolizumab + paclitaxel	mPFS: higher, acceptable safety profile	NCT04408118	[537]
VEGF/apatinib	Phase II	Advanced TNBC	Camrelizumab combined with apatinib	ORR: 43.3%, DCR: 63.3 vs. 40.0%, mPFS: 3.7 vs. 1.9 months	NCT03394287	[539]
VEGF/ENMD‐2076	Phase II	Locally advanced, metastatic TNBC	Aurora and ENMD‐2076	CBR: 16.7% (6‐months)	NCT01639248	[540]

#### 5.3.4 Inhibition of Signaling Pathways in TNBC

TNBC cells exhibit hyperactivation of several signaling pathways, including the PI3K/Akt/mTOR, Wnt/β‐catenin, JAK2/STAT3, Ras/Raf/MEK/ERK, Notch, and Hh pathways, all of which contribute to tumor initiation and progression [543]. Therefore, targeting these pathways is a promising therapeutic approach for TNBC.

Buparlisib (BKM120), an orally potent pan‐class I PI3K inhibitor, was assessed in a phase II trial (NCT01790932) of patients with mTNBC. The trial demonstrated a CBR of 12%, an mPFS of 1.8 months, and an mOS of 11.2 months [544]. Copanlisib is another potent pan‐class I PI3K inhibitor that has shown enhanced antitumor activity in TNBC. In a phase I/II trial (NCT04345913) evaluating eribulin in combination with copanlisib in mTNBC, the mPFS was found to be 6.95 versus 4 months for the control arm [545]. In the case of Akt inhibitors, the phase II PAKT trial (NCT02423603) evaluated capivasertib combined with paclitaxel versus placebo plus paclitaxel as the first‐line treatment for mTNBC. The mPFS was 9.3 months in the capivasertib group compared with 3.7 months in the placebo group [546]. Similarly, the randomized phase II LOTUS trial (NCT02162719) assessed ipatasertib plus paclitaxel versus placebo plus paclitaxel in patients with inoperable locally advanced or mTNBC. The mOS was numerically longer in the ipatasertib arm (25.8 months) compared with the placebo arm (16.9 months) [547]. The PATHFINDER trial (NCT04464174), a multicenter, open‐label, phase IIa study, investigated ipatasertib combined with nontaxane chemotherapy in previously treated advanced TNBC. The combination of ipatasertib with capecitabine, and eribulin demonstrated acceptable safety. Notably, the eribulin plus ipatasertib regimen showed promising activity independent of PI3K/Akt mutation status, warranting further investigation [548]. In the context of mTOR inhibitors, an open‐label phase II NECTAR trial (NCT01931163) investigated the combination of everolimus and cisplatin in patients with residual TNBC, following neoadjuvant chemotherapy. Among the 22 patients, five achieved RCB‐I at surgery, corresponding to a response rate of 23% [549]. The ongoing phase II FUTURE‐SUPER trial (NCT04395989) is a randomized, open‐label study that evaluates first‐line subtype‐based treatment strategies for TNBC. In this trial, everolimus combined with nab‐paclitaxel was tested in metastatic mesenchymal‐like tumors exhibiting PI3K pathway activation. The mPFS was 11.3 months in the subtype‐based treatment group compared with 5.8 months in the control group, showing a significant improvement [550].

For the Wnt/β‐catenin targeting pathway, an open‐label, multicenter phase I clinical trial (NCT01351103) evaluated the porcupine inhibitor WNT974 (LGK974), both as a monotherapy and in combination with the checkpoint inhibitor, spartalizumab. This treatment demonstrated manageable toxicity, but showed limited preliminary efficacy in patients with advanced solid tumors, including TNBC [551]. In addition, protein TK 7 (PTK7) is a Wnt pathway coreceptor expressed on the surface of various tumors and serves as the target for the antibody–drug conjugate (ADC) cofetuzumab pelidotin, which delivers an auristatin‐based microtubule inhibitor to tumor cells [552]. In a phase I clinical trial (NCT03243331) of gedatolisib in combination with cofetuzumab pelidotin in patients with mTNBC, the ORR was found to be 16.7% (three out of 18), the CBR at 18 weeks was 27.8%, and the mPFS was 2.0 months. Clinical benefits were observed predominantly in patients harboring genomic alterations in the PI3K and PTK7 pathways [552]. Tinengotinib (TT‐00420) is a selective small‐molecule kinase inhibitor that targets the JAK1/2 and Aurora/STAT3 pathways. A recently completed phase I trial (NCT03654547) demonstrated its high activity against TNBC, and it was well tolerated. In vitro studies using TNBC cell lines and in vivo experiments in a syngeneic model showed upregulation of *CXCL10* and *CXCL11* and reduced infiltration of TAMs following tinengotinib treatment [553]. Notch receptors are overexpressed in TNBC, and their activation requires cleavage of the Notch ligand–receptor complex by γ‐secretase (GS). GS inhibitors (GSIs) have emerged as promising agents that block the activation of all Notch receptors [554, 555]. A phase I trial (NCT01238133) investigated neoadjuvant chemotherapy combining the GSI RO4929097 with paclitaxel and carboplatin in TNBC patients [554]. The regimen showed promising activity, with a pCR rate of 50% and minimal residual disease observed in 30% of the cases. Another phase I study (NCT01876251) evaluated the GSI PF‐03084014 with docetaxel in advanced TNBC and reported an mPFS value of 4.1 months and significant clinical efficacy [555]. Table 7 presents the clinical trials assessing the efficacy of various signaling pathway inhibitors in TNBC treatment.

**TABLE 7:** Clinical trial assessing the efficacy of various signaling pathway inhibitors in TNBC treatment.

Target/drugs	Trial stages (phase)	Patients	Treatment	Result	NCT ID	References
PI3K/buparlisib	Phase II	Metastatic TNBC	Treated with buparlisib	CBR: 12%, mPFS: 1.8 months, and mOS: 11.2 months	NCT01790932	[544]
PI3K/copanlisib	Phase I/II	Metastatic TNBC	Eribulin combined with copanlisib	mPFS: 6.95 vs. 4 months	NCT04345913	[545]
Akt/capivasertib	PAKT (II)	Metastatic TNBC	Capivasertib + paclitaxel vs. placebo + paclitaxel	mPFS: 9.3 vs. 3.7 months	NCT02423603	[546]
Akt/Ipatasertib	LOTUS (II)	Locally advanced or metastatic TNBC	Ipatasertib + paclitaxel vs. placebo + paclitaxel	mOS: 25.8 vs. 16.9 months	NCT02162719	[547]
Akt/ipatasertib	PATHFINDER (IIa)	Advanced TNBC	Ipatasertib combined with nontaxane chemotherapies	Acceptable safety profile and promising activity	NCT04464174	[548]
mTOR/everolimus	NECTAR (II)	Residual TNBC	Everolimus with cisplatin	Response rate: 23%	NCT01931163	[549]
mTOR/Everolimus	FUTURE‐SUPER (II)	TNBC	Everolimus combined with nab‐paclitaxel	mPFS: 11.3 vs. 5.8 months	NCT04395989	[550]
Wnt/β‐catenin/WNT974	Phase I	TNBC	WNT974 with checkpoint inhibitor spartalizumab	Manageable toxicity but limited preliminary efficacy	NCT01351103	[551]
Wnt/β‐catenin/cofetuzumab pelidotin	Phase I	Metastatic TNBC	Gedatolisib in combination with cofetuzumab pelidotin	ORR: 16.7% (3/18), CBR at 18 weeks: 27.8%, and mPFS: 2.0 months	NCT03243331	[552]
JAK/STAT3	Phase I	TNBC	Tinengotinib	Upregulated *CXCL10/11* and reduced infiltration of TAMs	NCT03654547	[553]
NOTCH/RO4929097	Phase I	TNBC	RO4929097 with paclitaxel and carboplatin	pCR rate of 50% and minimal residual disease observed in 30% of cases	NCT01238133	[554]
NOTCH/PF‐03084014	Phase I	Advanced TNBC	PF‐03084014 with docetaxel	mPFS of 4.1 months and better clinical efficacy	NCT01876251	[555]

**FIGURE 9:**
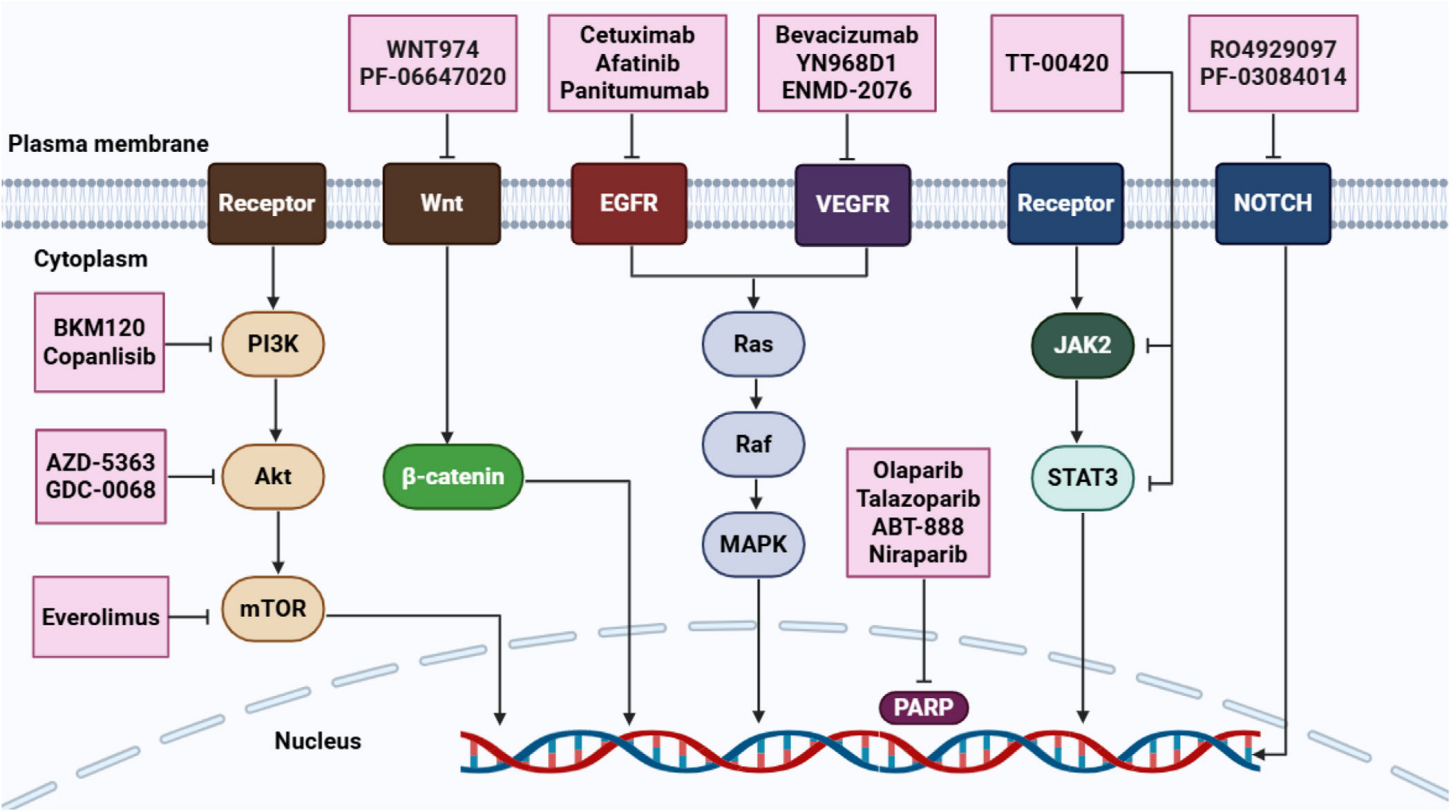
PARP, EGFR, VEGF, and signaling pathway inhibitors in TNBC treatment. Veliparib: ABT‐888, apatinib: YN968d1, buparlisib: BKM120, capivasertib: AZD5363, ipatasertib: GDC‐0068, cofetuzumab pelidotin: PF‐06647020, tinengotinib: TT‐00420. EGFR, epidermal growth factor receptor; PARP, poly(ADP‐ribose) polymerase; TNBC, triple‐negative breast cancer; VEGF, vascular endothelial growth factor. [Created using BioRender.com.]

Figure 9 illustrates the use of PARP, EGFR, VEGF, and various signaling pathway inhibitors in TNBC treatment.

## 6 Emerging Therapeutic Frontiers

### 6.1 ADCs Revolution

ADCs represent an innovative and predominant class of anticancer therapy that merges the targeting ability of antibodies with the potent cytotoxic effects of chemotherapeutic drugs [556]. By delivering drugs directly to cancer cells, ADCs enhance delivery precision while minimizing damage to healthy tissues and cells [557]. An ADC consists of three key components: an mAb, a linker, and a cytotoxic drug [558]. The linkers used in ADCs can be classified into two types: cleavable and noncleavable, both of which have been utilized in clinically approved or investigational ADCs [559]. Similarly, the cytotoxic agents in ADCs are mainly categorized into two groups: microtubule inhibitors and DNA‐damaging agents, each designed to disrupt cancer cell growth and survival [560]. The cytotoxic agents incorporated into ADCs provide mAbs with significantly enhanced cancer cell‐killing potency compared with conventional chemotherapy drugs [561]. They also help to improve drug resistance to other HER2‐targeted therapies by introducing a distinct mechanism of action, making them a valuable option in cancer treatment [562].

#### 6.1.1 Trastuzumab Emtansine

Trastuzumab emtansine (T‐DM1) is the first ADC to receive approval for solid tumors [563]. In 2013, the US FDA approved its use for treating HER2^+^ MBC, marking a significant advancement in the targeted cancer therapy [564].

It is composed of trastuzumab, a noncleavable thioether linker known as N‐maleimidomethyl cyclohexane‐1‐carboxylate, and DM1, a potent maytansinoid derivative that disrupts microtubules [565]. The mAb in T‐DM1 specifically targeted HER2, enabling selective drug delivery via receptor‐mediated endocytosis [566]. This targeted approach minimizes toxicity to healthy tissues and enhances treatment effectiveness of treatment [567]. T‐DM1 operates through several mechanisms, such as the targeted delivery of DM1 to HER2^+^ BC cells, trastuzumab‐mediated suppression of HER2 signaling, prevention of HER2 extracellular domain shedding, and activation of ADCC [568]. In the phase III EMILIA trial (NCT00829166), T‐DM1 demonstrated a longer mOS compared with capecitabine with lapatinib in patients with previously treated HER2^+^ ABC (29.9 vs. 25.9 months) [569]. Similarly, in the phase III TH3RESA trial (NCT01419197) in HER2^+^ MBC, T‐DM1 achieved a significantly longer mOS than treatment of physician's choice (22.7 vs. 15.8 months), with frequently reported adverse events in the T‐DM1 group [570]. In the phase III MARIANNE trial in HER2^+^ ABC, which compared T‐DM1 with or without pertuzumab against trastuzumab plus taxane, the mOS was comparable across arms (50.9, 53.7, and 51.8 months, respectively). However, among patients with an OR, mOS was longer with T‐DM1 (64.4 months) and T‐DM1 plus pertuzumab (not reached) compared with trastuzumab plus taxane (56.3 months) [571]. The phase III KATHERINE trial (NCT01772472) demonstrated the clinical benefit of T‐DM1 after neoadjuvant therapy for HER2^+^ BC, regardless of prior anthracycline‐ or nonanthracycline‐based chemotherapy [572]. In the phase III KAITLIN study (NCT01966471), T‐DM1 plus pertuzumab (AC‐KP) with taxane plus trastuzumab plus pertuzumab (AC‐THP) was administered after anthracycline‐based therapy in high‐risk HER2^+^ BC. After a median follow‐up of approximately 57 months in both arms, no significant difference in iDFS was observed in either the node‐positive subgroup or in the overall population, with 3‐year iDFS rates of 94.2% for AC‐THP and 93.1% for AC‐KP [573]. The most common adverse effects of T‐DM1 include anemia, fatigue, headache, increased serum aminotransferase levels, and thrombocytopenia [574, 575].

#### 6.1.2 Trastuzumab Deruxtecan

Trastuzumab deruxtecan (T‐DXd) is an ADC approved by the US FDA in 2019 for treating HER2^+^ MBC [576]. It consists of trastuzumab, a cleavable linker, and a cytotoxic topoisomerase I inhibitor that exhibits anticancer effect [577]. The conjugate remains stable in the plasma and, once inside the cells, releases the cytotoxic drug through lysosomal cathepsin cleavage [578]. This cleavability also facilitates a bystander killing effect, allowing the drug to target nearby tumor cells [579]. This distinguishes T‐DXd from T‐DM1 because T‐DXd has a higher antibody‐to‐drug ratio [580].

The action of T‐DXd involves two key mechanisms: trastuzumab inhibits HER2 signaling, while the deruxtecan part attaches itself to the topoisomerase I–DNA complex, causing DNA damage and triggering cell death [581]. In contrast to T‐DM1, T‐DXd has a release mechanism that enables it to cross the cell membranes more easily [582]. This characteristic allows it to produce a strong cytotoxic effect on nearby tumor cells, even if they do not express HER2 [583]. In the phase III DESTINY‐Breast03 trial (NCT03529110), T‐DM1 was compared with T‐DXd as a second‐line treatment for HER2^+^ ABC after progression to trastuzumab plus taxane. The mPFS was not reached with T‐DXd, whereas it was 6.8 months with T‐DM Additionally, the 12‐month OS rate was higher with T‐DXd (94.1%) than with T‐DM1 (85.9%) [584]. Commonly observed adverse events of T‐DXd treatment include nausea, vomiting, hair loss, decreased number of neutrophils, and respiratory problems [585, 586].

#### 6.1.3 Sacituzumab Govitecan

Trophoblast cell‐surface antigen (Trop‐2) is a transmembrane protein overexpressed in various cancer cells, is present in around 80% of BCs, and is associated with poor prognosis [587]. TROP‐2‐targeting ADCs employ mAbs that selectively bind to TROP‐2 receptors on tumor cells [588]. Trop‐2 expression has been reported in 93% of TNBC, 50% of ER^+^ BC, and 74% of HER2^+^ BC [589].

Sacituzumab govitecan (SG) is the first US FDA‐approved Trop‐2‐targeted ADC, showing notable efficacy in HR^+^/HER2^−^ and TNBC [590]. In the phase I/II IMMU‐132‐01 trial (NCT01631552), conducted in patients with HR^+^/HER2^−^ MBC progressing after ET and at least one prior chemotherapy, SG achieved an ORR value of 31.5%, with a median duration‐of‐response (mDOR) of 8.7 months, mPFS of 5.5 months, and mOS of 12 months at a median follow‐up of 11.5 months [591]. The phase III TROPiCS‐02 study (NCT03901339) demonstrated improved PFS and ORR with SG monotherapy in HR^+^/HER2^−^ MBC previously treated with ET and multiple chemotherapy lines [592]. Additionally, SG provided a mPFS of 5.5 versus 4.0 months with chemotherapy, with 6‐ and 12‐month PFS rates of 46 versus 30% and 21 versus 7%, respectively for endocrine‐resistant HR^+^/HER2^−^ ABC [593]. Moreover, OS results (LBA76) from TROPiCS‐02 showed a survival benefit with SG over physician's choice treatment (14.4 vs. 11.2 months), along with improved ORR, quality of life, and manageable safety in this heavily pretreated endocrine‐resistant HR^+^/HER2^−^ MBC [594].

In the phase I/II IMMU‐132‐01 trial (NCT01631552) involving 108 patients with TNBC, SG achieved an ORR of 33.3%, median response duration of 7.7 months, CBR of 45.4%, mPFS of 5.5 months, and mOS of 13.0 months [595]. Moreover, in the phase III ASCENT trial (NCT02574455), 468 TNBC patients with at least two prior lines and a taxane were randomized to receive SG or single‐agent chemotherapy (eribulin, vinorelbine, capecitabine, or gemcitabine). SG significantly improved mPFS (5.6 vs. 1.7 months) and mOS (12.1 vs. 6.7 months), with an ORR of 35 versus 5% for chemotherapy [596]. The NeoSTAR phase II study (NCT04230109) on response‐guided neoadjuvant SG in 50 patients with localized TNBC was mostly node‐negative (62%). After four cycles, 62% had a radiologic response, and 52% proceeded directly to surgery, achieving a pCR rate of 30%. The remaining patients received additional neoadjuvant chemotherapy, with six more achieving pCR [597]. Moreover, the phase II trial (NCT04039230) evaluating sequential SG and talazoparib in mTNBC, mPFS was 6.2 months, mOS was 18.0 months, ORR was 30.1%, and 6‐month CBR was 53.8% [598]. Common SG‐related adverse events include anemia, neutropenia, nausea, fatigue, alopecia, rash, and thrombocytopenia [597, 598].

### 6.2 Immunomodulation Strategies

Immunomodulatory strategies in BC focus on activating the immune system to target and eliminate cancer cells.  These strategies involve various approaches including the use of  immune checkpoint inhibitors, CTLA‐4 inhibitors, adoptive T‐cell therapy, and cancer vaccines.

#### 6.2.1 Immune Checkpoint Inhibitors

**FIGURE 10:**
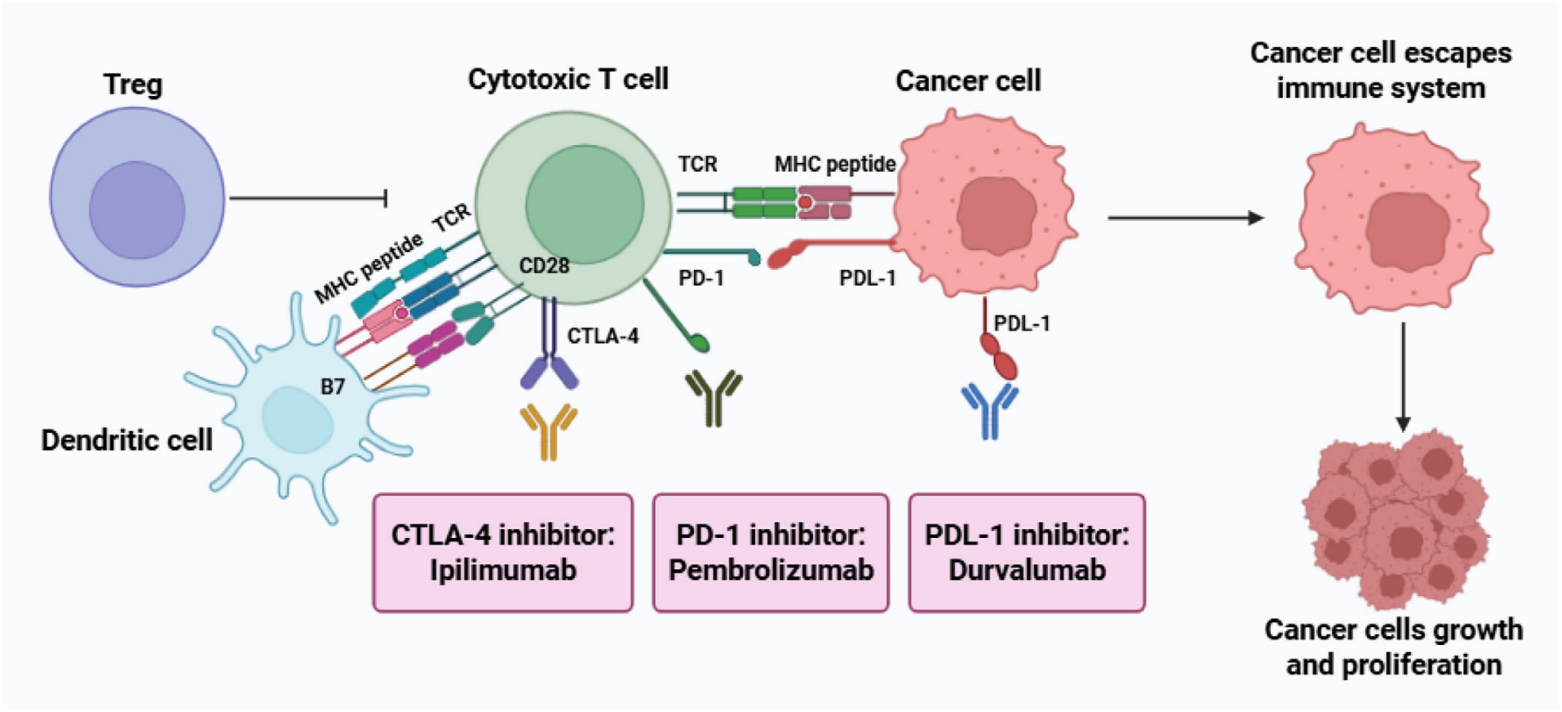
Immune escape in BC and therapeutic action of immune checkpoint inhibitors. In the TME, BC cells evade immune system by suppressing cytotoxic T‐cell activation through Treg cells and immune checkpoint pathways. Normally, B7 ligands on antigen‐presenting cells (dendritic cells) interact with CD28 on cytotoxic T‐cells, promoting T‐cell proliferation and immune activation. When B7 binds to CTLA‐4 on T‐cells, it inhibits their function and enhances Treg cell‐mediated immunosuppression. In addition, PD‐1, expressed on activated T‐cells, interacts with PD‐L1 on tumor cells, inducing T‐cell exhaustion and inhibiting antitumor responses. Monoclonal antibodies targeting these checkpoints (CTLA‐4, PD‐1/PD‐L1) can block inhibitory signals, reactivate T‐cells, and restore their ability to target cancer cells. BC, breast cancer; CTLA‐4, cytotoxic T‐lymphocyte antigen 4; MHC, major histocompatibility complex; PD‐1, programmed cell death‐1; PD‐L1, programmed cell death‐1 ligand; TCR, T‐cell receptor; TME, tumor microenvironment; Treg cells, regulatory T cells. [Created using BioRender.com.]

Immune checkpoint inhibitors function by blocking specific cell surface proteins that normally act as brakes on the immune system [599]. Immune checkpoints regulate both T cell activation and tolerance, ensuring immune balance and self‐tolerance under normal conditions. However, tumors can exploit these inhibitory pathways to evade the immune system [600]. In BC, the most critical checkpoints are the PD‐1/PD‐L1 axis and CTLA‐4, both of which suppress T‐cell activation [601]. mAb‐based therapies that target these checkpoints have been developed to restore antitumor immunity, and their therapeutic applications in BC are illustrated in Figure 10.

##### 6.2.1.1 Anti‐PD‐1/PD‐L1

Elevated PD‐1 expression in BC correlates with tumor aggressiveness. PD‐1 functions through its interaction with PD‐L1, expressed in around 20–30% of BC cases, with the highest levels found in TNBC [602]. Since PD‐1 is a coinhibitory receptor that suppresses T‐cell activity, blocking the PD‐1/PD‐L1 axis can restore T‐cell immunogenicity and enhance antitumor responses [603]. The combination of cemiplimab (anti‐PD‐1 suppressor) and REGN3767 (fully humanized antilymphocyte activation gene 3) mAb, combination showed enhanced anti‐PD‐1 effect in the I‐SPY2 phase II trial (NCT01042379) in high‐risk HER2^−^ EBC [604]. In addition, pembrolizumab (another PD‐1‐blocking mAb) has shown benefits in the KELLY trial (NCT03222856) when combined with eribulin for HR^+^/HER2^−^ MBC, with an mPFS of 6 months and a 1‐year OS of 59.1% [605]. The KEYNOTE‐355 phase III trial (NCT02819518) compared pembrolizumab plus chemotherapy with placebo plus chemotherapy in untreated, locally recurrent inoperable, or mTNBC. With a median follow‐up of approximately 26 months, patients with a combined positive score (CPS) ≥10 showed an mPFS of 9.7 months in the pembrolizumab group versus 5.6 months in the placebo group, with a greater benefit observed in PD‐L1‐enriched tumors [606]. Similarly, a phase III trial (NCT02555657) of the KEYNOTE‐119 study reported comparable OS between pembrolizumab and chemotherapy, although outcomes favored patients with CPS ≥10 tumors in TNBC [607]. Common anti‐PD‐1/PD‐L1 treatment‐related adverse events include neutropenia, anemia, alopecia, fatigue, diarrhea, asthenia, peripheral neuropathy, thyroid dysfunction, pneumonitis, and hepatitis [604, 607].

##### 6.2.1.2 Anti CTLA‐4

CTLA‐4 is a key immune checkpoint molecule expressed on T cells and Treg cells, where it elevates the activation threshold of T cells and suppresses antitumor immunity. Inhibition of CTLA‐4 with mAbs can enhance T‐cell effector activity and promote tumor cell‐killing efficiency. The two main antibodies that target CTLA‐4 in BC are tremelimumab and ipilimumab. Tremelimumab is a humanized IgG2 antibody which blocks CTLA‐4–B7 interactions to facilitate T‐cell activation [608]. A pilot trial combining tremelimumab with the anti‐PD‐L1 antibody durvalumab showed limited benefit in overall MBC, but TNBC patients responded more favorably. Surprisingly, even better outcomes were observed using durvalumab monotherapy [609]. Additionally, pembrolizumab combined with radiotherapy demonstrated an acceptable safety profile in a phase I study even with prolonged treatment [610]. Similarly, ipilimumab is another humanized IgG1 mAb targeting CTLA‐4, which has demonstrated notable antitumor activity in several cancers. In a multicenter phase II trial (NCT02834013) combining ipilimumab with nivolumab for unresectable or MBC, the ORR was 18%, with three out of 17 patients achieving objective responses, with mPFS and OS of 2 and 12 months, respectively, [611]. Despite these outcomes, CTLA‐4 inhibitors have shown limited efficacy in BC and are associated with severe immune‐related adverse effects, partly linked to the lysosomal degradation of CTLA‐4 [611].

#### 6.2.2 Adoptive T‐Cell Therapy

Adoptive T cell therapy has emerged as a powerful approach in cancer immunotherapy, significantly improving the recognition of tumor antigens capable of effectively eliminating cancer cells. This strategy encompasses multiple modalities, including TIL‐based therapy, engineered T‐cell receptor‐based therapy, chimeric antigen receptor (CAR)‐T cells, DC‐based therapies, and NK cell‐based therapies [612]. A phase I clinical trial (NCT05378464) is currently evaluating adoptive T cell therapy combined with a HER2‐pulsed DC vaccine and pepinemab/trastuzumab in patients with HER2^+^ MBC. The trial hypothesized that this combination may stimulate CD4^+^ HER2‐specific T‐cell responses, with safety and tolerability as the primary endpoints, and assessment of T‐cell immunity, immune subsets, and clinical efficacy as the secondary objectives [613].

CAR T‐cell therapy is one of the most advanced and effective forms of adoptive T‐cell therapy, harnessing the antigen specificity of antibodies to enhance the cytotoxic potential of T‐cells. This approach relies on the genetic modification of host T‐cells to express tumor‐targeting receptors or synthetic CARs, thereby promoting potent antitumor activity. CAR typically comprises extracellular single‐chain variable fragments, a transmembrane domain, immunoreceptor tyrosine‐based activation motifs, and costimulatory signaling elements. Preclinical studies have identified several promising BC antigens for CAR T cell therapy, including EGFR, FRα, HER2, integrin αvβ3, AXL, NKG2D, c‐Met, MUC1, mesothelin, ROR1, TROP2, and TEM8. Targets such as AXL, c‐Met, FRα, MUC1, mesothelin, NKG2D, and ROR1 have been successfully evaluated in both in vitro and in vivo models of TNBC. For instance, MUC1, which is expressed in over 90% of BC and 95% of TNBC cases, has been targeted using MUC28z‐CAR T cells that recognize aberrantly glycosylated MUC1, leading to antigen‐specific cytotoxicity and significant tumor reduction in xenograft models [614]. Similarly, EGFR is overexpressed in TNBC and has been explored as a CAR T‐cell target, demonstrating effective tumor inhibition with minimal toxicity to normal tissues both in vitro and in vivo study [615].

#### 6.2.3 Cancer Vaccines

Anticancer vaccines are designed to stimulate antigen‐specific T‐cell responses that can recognize and destroy tumor cells. The most studied vaccines are peptide‐based, derived from tumor‐associated antigens, while other forms include DC‐ and DNA‐based vaccines. Among the different vaccine types, peptide‐based vaccines have synthesis simplicity, cost effectiveness, and a favorable safety profile [616]. In patients with HER2^+^ BC (IHC 1+–3+), peptide vaccines showed a limited impact on DFS, even in those with HER2 overexpression [617]. Alternatively, glycolipid–peptide conjugate vaccines that activate NK T cells have been demonstrated to prevent lung metastasis in BC models, highlighting their potential as adjuvant therapies for high‐risk patients [618]. Moreover, DC‐based vaccines are another promising approach in which immature DCs isolated from patient blood can be stimulated with antigens and cytokines and then reinfused after maturation to trigger potent immune responses. Mature DCs significantly enhanced antigen‐specific CD8^+^ T‐cell activity against HER2 (4.5%; *p* < 0.005) and MUC1 (19%; *p* < 0.05), and induced a dose‐dependent cytotoxic T‐lymphocyte response (65%) capable of killing autologous BC cells in vitro, outperforming DCs loaded with autologous lysates (*p* < 0.005). Importantly, cryopreserved mature DCs retain their viability, functional phenotypes, and sterility [619]. An interventional study involving eight patients with ABC or MBC evaluated a DNA vaccine encoding a signaling‐deficient full‐length HER2/neu combined with low doses of IL‐2 and granulocyte‐macrophage colony‐stimulating factor. This vaccination induced strong humoral immune responses, though no marked improvement in T cell activity was observed [620]. In another approach, researchers developed a DNA vaccine targeting mammaglobin‐A (MAM‐A), a gene expressed exclusively in BC and overexpressed in 40–80% of cases. A phase I clinical trial demonstrated that this vaccine significantly increased both the number and frequency of MAM‐A‐specific CD8^+^ T cells, with early indications of prolonged PFS [621]. Moreover, a MUC1‐based mRNA vaccine demonstrated potent cytotoxic T‐lymphocyte activation against TNBC. When administered in combination with ipilimumab, an anti‐CTLA‐4 mAb, the mRNA vaccine elicited stronger T‐cell immunity than a single treatment [622].

### 6.3 Epigenetic Targeting

Epigenetics refers to heritable molecular processes regulated by environmental and cellular factors that influence gene expression without altering the actual DNA sequence. It controls chromatin dynamics through five major mechanisms namely DNA modification, histone modification, RNA modification, chromatin remodeling, and regulation by noncoding RNAs [623]. These mechanisms, along with their associated enzymes, transmit genetic information independently to DNA bases, and are crucial for normal development and physiological balance. However, disturbances in epigenetic regulation contribute significantly to cancer progression, including BC [624]. Epigenetic therapy is a promising approach that employs chemical agents to correct such alterations by targeting key epigenetic modulators. Established therapies focus on major epigenetic regulators, such as DNA methyltransferases (DNMTs), histone deacetylases (HDACs), histone methyltransferases, and histone demethylases [625]. Inhibitors of DNMTs and HDACs have shown therapeutic benefit in BC by reversing aberrant modifications and re‐establishing normal gene expression.

#### 6.3.1 DNMT Inhibitors

DNMT inhibitors (DNMTis) have emerged as a promising class of anticancer agents with notable antitumor potential in BC. By binding to DNMTs, these agents block DNA methylation and prevent silencing of tumor suppressor genes, thereby restoring their expression and restricting tumor progression [626]. Interestingly, DNMTis may also induce a *BRCA*‐mutant‐like state in *BRCA*‐wild type TNBC by promoting the mechanism of HRD [627]. The hypomethylating agents, azacitidine and decitabine, which primarily target DNMT1, were among the first epigenetic drugs approved for clinical use [623]. Although early clinical trials with these agents as monotherapies showed limited benefits, but preclinical and clinical data support their synergistic effects in combination therapies. For instance, a phase II trial using 5‐azacitidine and entinostat indicated a potential benefit in ABC, particularly in re‐sensitizing tumors to ET [628]. In addition, decitabine has been shown to reactivate silenced *BRCA1* in BC, enhance DNA repair, and sensitize tumors to chemotherapy. In TNBC, decitabine‐treated patient‐derived xenograft (PDX) organoids exhibited strong tumor growth inhibition at low doses [629]. Moreover, combined treatment with decitabine and eugenol suppresses CAF‐mediated invasion and angiogenesis, improving BC patient outcomes [626]. Compared with decitabine, olsalazine induced a greater upregulation of CDH1 while avoiding unwanted increases in uPA expression, thereby inhibiting EMT in MDA‐MB‐231 cells [630]. Liraglutide is a novel DNMTi that has shown antitumor activity in BC both in vitro and in vivo models. In combination with paclitaxel or methotrexate, liraglutide effectively reduce tumor growth and modulate *CDH1* and *ADAM33* expression through DNA demethylation [631].

#### 6.3.2 HDAC Inhibitors

HDAC inhibitors (HDACis) suppress the activity of HDACs, leading to increased histone acetylation and a relaxed chromatin structure. This facilitates access of TFs to DNA, thereby activating tumor‐suppressive genes [632]. In BC, HDACis slow cell proliferation and promote apoptosis, partly through the activation of genes, such as p21 [633]. Vorinostat was the first US FDA‐approved HDACi that demonstrated antitumor effects in TNBC by blocking nitric oxide‐driven histone deacetylation [634]. Additionally, apigenin in combination with vorinostat induced apoptosis in TNBC cells by modulating epigenetic regulators, apoptotic pathways and associated miRNAs [635]. Chidamide or tucidinostat is the first orally available selective HDACi that can target HDAC1, HDAC2, HDAC3, and HDAC10 and has demonstrated promising outcomes in BC therapy. In a phase III trial (NCT02482753), tucidinostat plus exemestane significantly improved mPFS (7.4 vs. 3.8 months with placebo) in postmenopausal patients with advanced HR^+^ BC, although treatment‐related adverse events were more in the combination group [636]. Moreover, clinical study following CDK4/6 inhibitor resistance showed a CBR of 6.8%, a mPFS of 2.0 months, and OS of 14 months in heavily pretreated HR^+^ MBC patients [637]. Furthermore, the use of tucidinostat plus exemestane as neoadjuvant therapy in the early stages of HR^+^/HER2^−^ BC produced encouraging clinical responses. The treatment was associated with adverse effects, such as anemia, thrombocytopenia, neutropenia, leukopenia, lymphopenia, hypoalbuminemia, and elevation of ALT [638].

## 7 Overcoming Therapeutic Resistance

### 7.1 Intratumoral Heterogeneity and Clonal Evolution

BC is a highly heterogeneous disease influenced by diverse genetic and nongenetic alterations that shape its clinical outcomes. Intratumoral heterogeneity (ITH) occurs at multiple levels, including genomic, transcriptomic, and proteomic levels and refers to the presence of cancer cells with distinct molecular profiles within the same tumor of a single patient [639]. The clonal evolution model, introduced by Nowell and based on Darwinian principles, suggests that successive generations of tumor cells acquire new mutations, with only the more adaptable variants surviving and proliferating, while less‐fit clones are lost [640]. Therefore, ITH and clonal evolution are key features of BC that drive its initiation, progression, and therapeutic resistance [641]. Notably, in HER2‐targeted therapies, approximately 5% of HER2‐amplified BC cases showed heterogeneous *HER2* gene amplification [639]. Additionally, researchers have performed a comprehensive genomic analysis of HER2^+^ BC, investigating both HER2‐amplified and nonamplified regions. Their findings revealed that the non‐HER2‐amplified components possess additional driver mutations, indicating that genetic ITH may contribute to resistance against targeted therapies [642]. Metabolic reprogramming further supports resistance mechanisms, as one study demonstrated that targeting fatty acid synthase or using metformin can overcome resistance to HER2‐targeted treatments by modulating metabolic heterogeneity [643, 644]. Moreover, analyses of patient samples before and after neoadjuvant chemotherapy with bevacizumab showed marked posttreatment transcriptomic alterations, suggesting their role in resistance [645]. A similar finding was observed in TNBC, in which neoadjuvant therapy led to the emergence of new subclones, reflecting enhanced ITH [646].

Multiple strategies have been proposed to address the role of ITH in therapeutic resistance, including combination therapies, adaptive treatment strategies, TME targeting, and the use of multiomics technologies. In HER2^+^ BC, combining mAbs, TKIs, and ADCs has shown the potential to counteract the reduced drug efficacy caused by ITH [647]. Adaptive therapy involves adjusting the treatment in response to tumor evolution and resistant clones, such as adding low‐dose verapamil and 2‐deoxyglucose during doxorubicin intervals to suppress glycolytic activity in BC and limit tumor adaptability driven by ITH [648, 649]. Advances in targeting BC stem cells (BCSCs) also hold promise; for instance, TNBC stem cells with elevated FXYD3 expression has shown sensitivity to Na+/K+ pump inhibitors [650]. Moreover, the EMSY protein has been identified as a key regulator of methionine metabolism, remodeling the TME to promote BCSC expansion and tumor growth. Thus, blocking EMSY‐driven metabolic pathways represents a potential therapeutic approach for TNBC [651]. Targeting TME offers another approach for addressing ITH. Treg cells, which play a central role in tumor immune evasion, are distributed within the TME, and selectively targeting Treg cells may help to overcome intratumoral Treg cell‐related heterogeneity and enhance immunotherapy [652]. Moreover, TAMs also contribute to ITH, as revealed by scRNA‐seq studies that showed significant intercellular diversity [653]. TAMs support immunosuppression and angiogenesis, but inhibit specific subsets, such as Trem2+Reg–TAMs, which have shown to boost immunotherapy efficacy, suppress tumor progression, and enhance T‐cell function [654]. Similarly, combining VEGF inhibitors with angiopoietin‐2 inhibitors can restrict Ang‐TAM recruitment, thereby overcoming resistance to antiangiogenic therapy and reducing TAM ITH [655]. Furthermore, multiomics approaches combined with machine learning (ML) can identify gene expression patterns linked to ITH and shown strong predictive potential, although their clinical validation requires further investigation [639].

### 7.2 Adaptive Bypass Signaling (e.g., FGFR1 Upregulation Post‐CDK4/6i)

Fibroblast growth factor/fibroblast growth factor receptor (FGF/FGFR) signaling plays an important role in several cancer‐related processes including cell proliferation, differentiation, migration, and survival [656]. Dysregulation of the FGF/FGFR pathway is commonly observed in various cancers, and most studies have focused on FGFR1, FGFR2, and FGFR3 modulation. Among them, *FGFR1*, located on chromosome 8p11‐12, is frequently altered and mutated in approximately 15% BC [657]. Importantly, *FGFR1* overexpression drives resistance to CDK4/6i used alone or in combination with ET [658]. Furthermore, *FGFR1* overexpression not only contributes to therapeutic resistance, but is also associated with poor clinical outcomes. In addition, *FGFR1* overexpression enhances cancer cell stemness and activates Akt/Erk–ER signaling, promoting palbociclib resistance in luminal A BC cells [659]. In the MONALEESA‐2 trial of ribociclib, circulating tumor DNA (ctDNA) analysis revealed that patients with *FGFR1* amplification had shorter PFS than those carrying wild‐type *FGFR1*. Specifically, *FGFR1*‐amplified patients treated with ribociclib plus letrozole had an mPFS of 22 months, whereas patients without amplification did not reach mPFS even after 32 months of follow‐up, indicating a more favorable outcome [660]. In the PALOMA‐3 trial, early disease progression was associated with risk factors, such as *FGFR1* amplification, *TP53* mutations, and high plasma tumor content in ER^+^ ABC [661].

*FGFR1* amplification also contributes to resistance to ER, PI3K, and CDK4/6 inhibitors, suggesting that mTOR inhibitors may offer therapeutic benefits in ER^+^/FGFR1^+^ MBC [662]. Although previous attempts to combine FGFR inhibitors with ET in HR^+^ MBC did not significantly influence clinical practice, ongoing studies are exploring the potential of combining FGFR inhibitors with CDK4/6 inhibitors and ET [663]. For instance, combined inhibition of ER, CDK4/6, and FGFR1 has shown to overcome resistance to both ET and palbociclib in luminal BC [664]. In a phase II trial (NCT01528345), patients with FGFR pathway amplification were treated with fulvestrant plus either dovitinib (a multikinase inhibitor targeting FGFR1/3) or placebo. The dovitinib arm showed an improved mPFS of 10.9 months compared with 5.5 months in the control group [665]. Similarly, the NCI‐MATCH phase II study (NCT02465060) assessed AZD4547 monotherapy in 48 patients with *FGFR* amplifications or mutations, including 13 patients with BC. The median PFS was 3.4 months, with a 6‐month PFS rate of 15%. Among patients with FGFR fusions, the response rate was 22% and the 6‐month PFS rate was 56%. However, the study did not achieve the predefined 16% ORR across all *FGFR* alterations, which likely reflects their biological heterogeneity [666]. Additionally, the combination of palbociclib, fulvestrant, and the pan‐FGFR inhibitor erdafitinib is being evaluated in a phase Ib trial (NCT03238196) for patients with HR^+^/HER2^−^ MBC harboring *FGFR1–4* amplification. However, this regimen showed limited tolerability, with several patients discontinuing treatment due to safety concerns, and the clinical outcomes were modest with an mPFS of 3 months and a 6‐month CBR of 28% [667]. Furthermore, rogaratinib is under investigation in combination with palbociclib and fulvestrant for treating HR^+^/FGFR1/2^+^ BC [668]. Phase I ROGABREAST trial (NCT04483505) assessed this triplet strategy (rogaratinib, fulvestrant, and palbociclib) in HR^+^ ABC with *FGFR1/2* amplification or overexpression. The results showed an mPFS of 113 days, with 44% of the patients maintaining PFS beyond 180 days. These findings suggest that the combined inhibition of FGFR1/2, CDK4/6, and ER is tolerable at standard doses and may offer clinical benefit in second‐line HR^+^/FGFR1/2^+^ BC progression after CDK4/6 inhibitor plus AI therapy [669].

### 7.3 Liquid Biopsy for Resistance Mutation Detection (ESR1, PIK3CA)

Liquid biopsy (LB) is a minimally invasive technique that examines ctDNA, cell‐free DNA, circulating tumor cells (CTCs), extracellular vesicles, and other tumor‐derived components in body fluids [670]. It has emerged as a valuable tool for BC management and offers multiple clinical applications. LB supports early cancer detection, often before symptoms appear or imaging reveals tumors, which is particularly beneficial for high‐risk groups where timely intervention can alter disease progression [671]. It also provides real‐time monitoring of treatment response, allowing therapy adjustments based on the dynamic genetic changes inside the tumor cells [672]. Several molecular biomarkers have gained clinical relevance through LB, including *PIK3CA* and *ESR1* mutations, both linked to disease progression and therapy resistance in MBC [670, 673]. *PIK3CA* mutations, present in up to 40% of HR^+^/HER2^−^ MBC cases, primarily occur in exon 9 (E545K, E542K) and exon 20 (H1047R), while *ESR1* mutations commonly affect the ligand‐binding domain (D538G, Y537S, Y537N, Y537C, E380Q) and are a frequent cause of acquired endocrine resistance in ER^+^/HER2^−^ MBC [674].

Recent investigations have applied LB for the detection of *PIK3CA* and *ESR1* mutations in BC. One study introduced a highly sensitive and specific multimarker liquid bead array assay capable of simultaneously identifying hotspot mutations in both genes. This assay reliably detected mutation‐allelic‐frequencies as low as 0.1%, revealing *PIK3CA* mutations in 13.6% and *ESR1* mutations in 72.7% of single CTCs [674]. Another study reported actionable *ESR1* and *PIK3CA* alterations in 42% of BC patients using LB, including *PIK3CA* alterations in 27% (H1047×12%, E545×8%, E542K 5%), and *ESR1* mutations in 24% (D538G, 11%; and Y537S, 8%). Comutations were found in 9% of cases, whereas multiple *ESR1* and *PIK3CA* variants were observed in 13 and 10% of mutation‐positive patients, respectively. Analysis of ctDNA further confirmed the feasibility of integrating LB into molecular pathology, offering high sensitivity, specificity, and real‐time insights to guide personalized therapy [675]. Another analysis identified *ESR1* mutations in 25.9% and *ERBB2* mutations in 3.7% of cases using LB, suggesting therapy modifications, such as the use of fulvestrant, elacestrant, or neratinib in MBC. Collectively, these findings highlight LB as a noninvasive and reliable tool for refining therapeutic strategies and improving outcomes in MBC [676].

Elacestrant, an US FDA‐ and EMA‐approved oral selective SERD, is an effective therapeutic option for patients with *ESR1*‐mutated tumors [677]. A large multicenter study involving nearly 6000 patients utilized novel next‐generation sequencing and digital droplet PCR assays to detect *ESR1* mutations in both tissue and LB samples, emphasizing the importance of developing highly sensitive methods for early mutation detection to guide elacestrant‐based treatment decisions [678]. The phase III EMERALD trial (NCT03778931) demonstrated that elacestrant significantly improved PFS compared with standard therapies in previously treated ER^+^/HER2^−^ ABC with *ESR1* mutations, leading to its regulatory US FDA approval for ET‐resistant patients [679]. Meanwhile, the phase III PADA‐1 trial (NCT03079011) represented the first prospective study to evaluate treatment switching based on b*ESR1* ^mut^ (*ESR1* mutation in blood) detection in ER^+^/HER2^−^ MBC, showing a marked PFS advantage with fulvestrant (11.9 months) over continued AI therapy (5.7 months) [680]. Additionally, *PIK3CA* mutations identified in LB have been linked to reduced sensitivity to CDK4/6 inhibitors in MBC. At baseline, patients harboring these mutations showed a significantly shorter median PFS (7.44 vs. 12.9 months) when treated with CDK4/6 inhibitors plus ET. This highlights the need to establish optimal treatment sequencing strategies for *PIK3CA*‐mutant BC [681]. Camizestrant (AZD9833) is a next‐generation oral SERD that has demonstrated encouraging results in preclinical ER^+^ BC models, both as monotherapy and in combination with CDK4/6 or PI3K/Akt/mTOR inhibitors, effectively addressing endocrine and CDK4/6 inhibitor resistance [682]. While *PIK3CA* mutations exert variable effects on PI3Kα‐selective compared with pan‐PI3K inhibitors, resistance caused by *PIK3CA* mutations can potentially be overcome with the novel allosteric pan‐mutant‐selective PI3Kα inhibitor RLY‐2608 [683].

## 8 Future Perspectives

### 8.1 Artificial intelligence‐Driven Drug Discovery and Biomarker Identification

Artificial intelligence (AI) has revolutionized modern oncology by providing advanced tools for cancer care and research. In drug discovery, AI has transformed the traditional lengthy and costly process of developing new therapies [684]. Its application enhances drug efficacy, reduces toxicity, and enables the advancement of molecular modeling, simulation methods, and the identification of new antitumor agents and antibodies [685]. Additionally, AI plays a crucial role in uncovering the mechanisms of drug toxicity and advancing precision medicine, thereby accelerating the design and delivery of targeted cancer therapies [686].

In early‐stage drug discovery, AI‐driven approaches enhance target identification, virtual screening, and molecular docking, enabling accurate predictions that accelerate the selection of potential drugs. It also contributes to de novo drug design and lead optimization, where advanced algorithms generate novel molecular structures and refine their properties to improve both safety and effectiveness [685]. In addition, the integration of AI and ML has significantly advanced cancer research by enabling the analysis of large‐scale genomic, proteomic, and clinical datasets to predict potential therapeutic targets [687]. More recently, AI has been applied to investigate drug resistance, using its powerful data processing and pattern recognition abilities to extract insights from clinical and omics data, predict resistance patterns/mechanisms, and assist in the development of innovative strategies to overcome drug resistance [688].

In cancer research, AI has emerged as a powerful tool for biomarker discovery and has greatly advanced diagnosis and prognosis. By analyzing large and complex datasets, AI can identify biomarker signatures that enhance early detection and personalized treatment strategies [689]. In addition, it has enhanced cancer imaging and diagnostics by improving the accuracy of detection, classification, and outcome prediction [687]. Different fields, such as pathology, radiology, and medical imaging, are now getting benefitted from AI algorithms, which is capable of interpreting diverse imaging modalities, with notable success in improving BC diagnosis through mammography and biopsy analysis [690]. Beyond diagnostics, AI contributes to clinical trial design by streamlining patient recruitment and stratification, thereby lowering costs and accelerating the timelines [686]. AI has become integral to BC management, as it supports early diagnosis, risk prediction, treatment planning, therapy response assessment, drug discovery, and prognosis [691]. In medical imaging, AI enhances cancer detection in mammography, MRI, and ultrasound, achieving accuracy comparable to that of expert radiologists. In pathology, it improves biomarker evaluation, including *HER2* and *Ki67*, and contributes significantly to the management of TNBC through improved prognosis and subtype classification [692]. ML and deep learning (DL) have further transformed BC research, ML enables precise diagnosis through image analysis, strengthening survival prediction, and refining risk assessment, whereas DL methods, such as convolutional neural networks, allow accurate tumor detection and classification from mammograms and MRI scans [693]. Recently, transcriptomic data combined with ML have been applied for biomarker discovery and biosensor development, offering a pipeline to identify genetic markers for classifying BC into nonmalignant, nontriple‐negative, and triple‐negative groups [694]. Despite these advancements, integrating AI into clinical practice and precision medicine still faces challenges, including the need for better regulation, transparency, fairness, and alignment with clinical workflows [691].

### 8.2 Patient‐Derived Organoid Models for Personalized Therapy

Patient‐derived organoids (PDOs) are three‐dimensional structures generated from a patient's tumor cells that closely mimic the histological features, genetic diversity, and functional characteristics of the original tumor [695]. Unlike conventional cell lines and PDXs, PDOs preserve the cellular complexity and internal heterogeneity of cancer tissues, making them highly valuable for translational cancer research and personalized therapy [696]. They have shown strong predictive value, which allows reliable prediction of patient‐specific drug responses, showing a strong correlation between PDO drug testing and clinical outcomes [697]. The establishment of PDO biobanks further supports drug discovery, screening, safety evaluation, and exploration of tumor‐specific molecular mechanisms [698]. Serving as advanced preclinical models, PDOs enable gene editing, molecular profiling, biomarker discovery, and therapeutic testing, highlighting their potential to advance precision oncology and improve individualized patient care [699].

PDOs are advanced in vitro models that replicate the TME, gene expression patterns, and cellular composition of cancers, including BC, and have great potential in drug discovery, prognosis, and personalized therapy [700]. In BC, PDOs serve as a real‐time platform for evaluating tailored treatments, particularly for refractory cases [701]. Research has shown that PDOs can predict microtubule‐targeting drug sensitivity, correlating with improved distant RFS in patients receiving adjuvant chemotherapy, thereby guiding personalized treatment strategies for advanced BC [701]. It also preserve the histological architecture of tumors and demonstrate patient‐specific responses to chemotherapy in BC [702]. Importantly, PDOs provide insights into patient‐specific tumor evolution. In one study, organoids derived from pre and posttreatment samples (O‐PRE and O‐POST) revealed higher proliferation rates in O‐POST samples, marked by elevated Ki67 levels, and adopted a more aggressive stem‐like phenotype. This was evident through increased CD24^low^/CD44^low^ and EPCAM^low^/CD49f^high^ cell populations, indicating enhanced tumor‐initiating and metastatic capacities, particularly in invasive lobular carcinoma. Molecular analysis further revealed decreased HER2 expression and increased EGFR expression in the O‐POST models [696]. Furthermore, PDOs preserve ITH in BC, allowing transcriptomic analyses to capture the biological complexity of the original tumor [703]. Combining PDOs with CTC models offers a powerful approach to study ITH and solve the challenges, representing a potential paradigm shift toward advanced personalized treatment strategies for MBC [704].

MBC‐PDOs have recently been developed as reliable tools for drug screening, offering representative models that capture the features of drug‐resistant and‐metastatic tumors. These organoids retain the characteristics of their original samples and hold great value in preclinical research for therapeutic decision guidance [705]. A mechanically induced 3D organoid platform developed from ER/PR/HER2^+^ BC provides deeper insights into tumor biochemistry, cellular composition, and molecular mechanisms, supporting the design of personalized treatment strategies [706]. TNBC‐PDOs have proven to be robust systems for studying tumor biology, heterogeneity, and progression at the single‐cell level, aiding in the development of personalized treatment strategies [707]. They are widely applied for drug sensitivity testing, drug screening, reflecting patient‐specific treatment responses, and demonstrated predictive value for survival outcomes in TNBC [708]. A case study further highlighted their clinical utility: in stage IV BC, PDO‐based drug testing identified sensitivity to SG, which achieved a strong pathological response and improved prognosis [709]. Similarly, PDO‐guided treatment of mTNBC with liver metastases resulted in a partial response, demonstrating the potential of PDOs in TNBC [710]. Additionally, a novel TNBC‐PDOs model was developed from patient samples after neoadjuvant chemotherapy, offering a promising platform to explore molecular alterations and identify personalized therapeutic options for patients with residual disease [711]. Furthermore, preclinical studies using TNBC‐PDOs and xenografts have validated the therapeutic potential of WEE1 inhibitors, providing a cost‐ and time‐efficient approach for personalized therapy [712]. Moreover, PDO‐based drug screening has identified emodin and platycodin D as promising candidates with broad efficacy across multiple BC subtypes, highlighting their potential for future therapeutic development [713].

### 8.3 Clinical Trial Designs (Basket Trials, Adaptive Platforms)

Clinical trial designs in BC research have evolved significantly, shifting toward more precise and efficient approaches. Among these, basket trials have emerged as a promising strategy for BC treatment, enabling the evaluation of novel therapies across multiple tumor types that share specific genetic alterations, including rare mutations [714]. Unlike traditional trials, which classify patients based on tumor histology or anatomical location, basket designs focus on the underlying molecular profiles, thus focusing on the development of targeted therapies for BC with distinct genetic abnormalities [715]. For example, a phase II basket study (NCT02454972) investigated lurbinectedin, a selective inhibitor of oncogenic transcription in patients with pretreated germline *BRCA1/2* MBC. The results demonstrated a mDoR of 8.6 months, mPFS of 4.1 months, and mOS of 16.1 months, with a predictable and manageable safety profile [716]. In addition, SKB264 (MK‐2870), a novel anti‐TROP2 ADC, was evaluated in a phase I/II single‐arm basket trial (NCT04152499) involving previously treated patients with HR^+^/HER2^−^ MBC. The study reported a manageable safety profile and encouraging antitumor activity, with an ORR of 36.8%, DCR of 89.5%, mDoR of 7.4 months, 6‐month DoR rate of 80%, mPFS of 11.1 months, and a 6‐month PFS rate of 61.2% [717]. Additionally, a phase II basket study (NCT04579380) assessed tucatinib plus trastuzumab in HER2‐mutated MBC, showing a mDoR of 12.6 months, DCR of 80.6%, mPFS of 9.5 months, and mOS of 20.1 months [718]. Furthermore, the SUMMIT phase II basket trial (NCT01953926) investigated neratinib alone or in combination with trastuzumab in HER2‐mutant and mTNBC, demonstrating promising clinical efficacy supported by genomic analysis [719].

Platform trials differ from basket trials in that they test multiple hypotheses within a single protocol, with designs that can vary widely. A key innovation in platform trials is adaptive design, which allows protocol modifications based on interim data analyses [720]. These designs often employ mini‐randomizations using Bayesian methods, enabling dynamic allocation of patients to treatments, showing greater promise while discontinuing ineffective options [721]. This flexibility improves trial efficiency and optimizes patient outcomes by integrating individual‐level information [720]. For example, the I‐SPY 2 trial is an ongoing multicenter, open‐label, adaptive phase II platform study of high‐risk BC. I‐SPY 2 evaluated multiple investigational agents alongside standard neoadjuvant therapy, offering a unique framework to test diverse treatments within a single trial, and generating valuable insights into BC management [722]. In addition, in the I‐SPY2 phase II adaptive trial, the combination of durvalumab, olaparib, and paclitaxel (DOP regimen) was evaluated in high‐risk stage II/III HER2^−^ BC. The DOP demonstrated superior efficacy compared with standard neoadjuvant chemotherapy, particularly in a highly responsive subgroup of HR^+^/HER2^−^ patients [723]. Similarly, the I‐SPY2 adaptive platform evaluated ado‐trastuzumab emtansine plus pertuzumab (T‐DM1/P) against paclitaxel, trastuzumab, and pertuzumab (THP) in HER2^+^ BC. Analyses revealed that pretreatment HER2 pathway signaling and phosphorylation correlated with response to both regimens, suggesting that such biomarkers may help identify patients who can safely receive less cytotoxic chemotherapy without compromising outcomes [724]. Additionally, the IND.241 trial (NCT05601440), conducted by the Canadian Cancer Trials Group, is a liquid‐biopsy‐informed platform study investigating treatment strategies for CDK4/6 inhibitor‐resistant ER^+^/HER2^−^ MBC. This master protocol design integrated clinical data, genomics, and radiomics across sub‐studies, facilitating the discovery of biomarkers for response, resistance, and disease progression [725].

## 9 Conclusions

Breast cancer remains a major global health concern and is characterized by its heterogeneous nature and complex etiology. It presents with diverse clinical and molecular profiles and is classified into distinct subtypes based on tumor origin, progression, and biomolecular features. Numerous signaling pathways are critically involved in BC development and progression, including ER, HER2, PI3K/Akt/mTOR, Wnt/β‐catenin, JAK2/STAT3, Notch, and Hh. These pathways contribute significantly to tumor proliferation, survival, and metastasis. Despite extensive preclinical research and clinical trials, there is still no definitive therapy that is capable of consistently achieving optimal outcomes across all BC subtypes. Challenges, such as heterogenicity, drug resistance and disease recurrence, continue to hinder long‐term treatment success. Therefore, the selection of effective therapeutic regimens requires careful evaluation of molecular and clinical variables. In response to these challenges, the development of targeted therapies has emerged as a key strategy for the personalized management of BC.

Targeted treatments, including mAbs, TKIs, ADCs, PI3K/Akt/mTOR pathway inhibitors, CDK4/6 inhibitors, PARP inhibitors, EGFR inhibitors and antiangiogenic agents have reshaped BC treatment paradigms. These therapies have been evaluated both as monotherapies and in combination with conventional treatments, leading to several receiving regulatory approval for their clinical use. In HER2^+^ BC, mAbs, such as trastuzumab, pertuzumab, and margetuximab, are often combined with chemotherapy. TKIs, including lapatinib, neratinib, tucatinib, and pyrotinib, are integrated with endocrine and chemotherapeutic agents to enhance their efficacy. Moreover, ADCs, such as T‐DM1 and T‐DXd, are employed as second‐line therapies and are currently under investigation in combination regimens involving immune checkpoint inhibitors, CDK4/6 inhibitors, and other TKIs. For HR^+^/HER2^−^ BC, CDK4/6 inhibitors, such as palbociclib, ribociclib, abemaciclib, and dalpiciclib, are commonly used alongside ET. Similarly, PI3K/Akt/mTOR inhibitors, including alpelisib, inavolisib, taselisib, capivasertib, ipatasertib, everolimus, and gedatolisib, have been administered to HR‐positive patients. Antiangiogenic agents like bevacizumab are combined with paclitaxel or capecitabine, while ramucirumab has been evaluated with docetaxel. In *BRCA*‐mutant TNBC, PARP inhibitors, such as olaparib, talazoparib, veliparib, and niraparib, are used as precision therapies. Moreover, EGFR inhibitors, including cetuximab and panitumumab, and VEGF inhibitors, including bevacizumab and apatinib, have shown promise against TNBC. Nevertheless, the clinical application of targeted therapies for TNBC remains limited owing to its intrinsic heterogeneity, aggressive nature, and high recurrence rates. Advancing TNBC treatment requires more innovative and individualized approaches that integrate genetic, clinical, and molecular data to optimize therapeutic precision, safety, and efficacy. The current lack of durable responses underscores the urgent need for ongoing translational and clinical research focused on personalized therapeutic strategies for TNBC.

In summary, BC is one of the most prevalent and extensively studied malignancies in the world. Although significant progress has been made in understanding the molecular landscape and developing targeted treatments, continued research is imperative. Future research should focus on novel approaches that can address resistance, improve efficacy, and enhance patient outcomes.

## Author Contributions

Md Abdus Samad, Iftikhar Ahmad, Mohammad Rashid Khan, and Mohd Suhail conceived, designed, and performed the literature survey and drafted the article's first draft. Mohd Shahnawaz Khan, Torki A. Zughaibi, Khaled Alhosaini, Iftikhar Ahmad, Ajoy Kumer, and Shams Tabrez finalized the content and provided inputs for further improvement. All authors have accepted responsibility for the entire content of this manuscript and approved its submission.

## Ethics Statement

The authors have nothing to report.

## Conflicts of Interest

The authors declare no conflicts of interest.

## Data Availability

The authors have nothing to report.
